# A continuous-time MaxSAT solver with high analog performance

**DOI:** 10.1038/s41467-018-07327-2

**Published:** 2018-11-19

**Authors:** Botond Molnár, Ferenc Molnár, Melinda Varga, Zoltán Toroczkai, Mária Ercsey-Ravasz

**Affiliations:** 1 Faculty of Physics, Babeş-Bolyai University, Cluj-Napoca, 400084 Romania; 2 Faculty of Mathematics and Computer Science, Babeş-Bolyai University, Cluj-Napoca, 400084 Romania; 3 Transylvanian Institute of Neuroscience, Cluj-Napoca, 400157 Romania; 4 Department of Physics, University of Notre Dame, Notre Dame, IN 46556 USA; 5 Center for Vascular Biology Research and Department of Medicine, Beth Israel Deaconess Medical Center, Boston, MA 02215 USA; 6 Romanian Institute of Science and Technology, Cluj-Napoca, 400487 Romania

## Abstract

Many real-life optimization problems can be formulated in Boolean logic as MaxSAT, a class of problems where the task is finding Boolean assignments to variables satisfying the maximum number of logical constraints. Since MaxSAT is NP-hard, no algorithm is known to efficiently solve these problems. Here we present a continuous-time analog solver for MaxSAT and show that the scaling of the escape rate, an invariant of the solver’s dynamics, can predict the maximum number of satisfiable constraints, often well before finding the optimal assignment. Simulating the solver, we illustrate its performance on MaxSAT competition problems, then apply it to two-color Ramsey number *R*(*m*, *m*) problems. Although it finds colorings without monochromatic 5-cliques of complete graphs on *N* ≤ 42 vertices, the best coloring for *N* = 43 has two monochromatic 5-cliques, supporting the conjecture that *R*(5, 5) = 43. This approach shows the potential of continuous-time analog dynamical systems as algorithms for discrete optimization.

## Abstract (web summary)

Continuous-time computation paradigm could represent a viable alternative to the standard digital one when dealing with certain classes of problems. Here, the authors propose a generalised version of a continuous-time solver and simulate its performances in solving MaxSAT and two-colour Ramsey problems.

## Introduction

Digital computing, or Turing’s model of universal computing is currently the reigning computational paradigm. However, there are large classes of problems that are apparently intractable on digital computers, requiring resources (time, memory, and/or hardware) for their solution that scale exponentially in the input size of the problem (NP-hard)^1^. Such problems, unfortunately, are abundant in sciences and engineering, for example, the ground-state problem of spin-glasses in statistical physics^2,3^, the traveling salesman problem^4^, protein folding^5^, bioinformatics^6^, medical imaging^7^, scheduling^8^, design debugging, Field Programmable Gate Array routing^9^, probabilistic reasoning^10^, etc. It is believed that in order to make progress on solving such problems one might have to look beyond computation with digital Turing machines. Analog computing and quantum computing present two promising and possibly revolutionary approaches, complementing complementary metal–oxide–semiconductor technology^11^ in solving certain types of hard computational problems. However, quantum computing currently faces fundamental physics and engineering challenges that still need to be solved^12,13^, leaving analog computing as a possibly more feasible option^14,15^. Although explored in the 1950s, it was abandoned in favor of the digital approach, due to the technical challenges it posed (for a historical survey see ref. ^16^). By now, however, technology has matured enough to control much better the physics at the small-scale, making it worthwhile revisiting analog computing, at least at an application-specific level. Accordingly, increasing effort is being dedicated to both problem-driven^17–29^ but also general purpose analog computing^30–32^, including analog computability theory^32–34^.

One quintessential family of intractable problems that could potentially be tackled with special-purpose analog devices are Boolean satisfiability problems, both in their decision (SAT) and optimization forms (MaxSAT). In SAT we are given a set of *M* logical clauses in conjunctive normal form (CNF), *C*_1_, *C*_2_, …, *C*_*M*_ over Boolean variables *x*_1_, …, *x*_*N*_, *x*_*i*_ ∈ {0, 1}. Typically, one studies *k*-SAT problems where every clause involves *k* literals (a literal is a variable or its negation). The task is to set the truth values of all the variables such that all the clauses evaluate to TRUE (“0” = FALSE, “1” = TRUE). It is well known that *k*-SAT with *k* ≥ 3 is NP-complete and thus any efficient solver for 3-SAT implies an efficient solver for all problems in the NP class (Cook-Levin theorem, 1971)^8,35,36^. The NP class is the set of all decision-type problems where one can check in polynomial time the correctness of a proposed solution (but finding such a solution can be exponentially costly).

MaxSAT (or Max *k*-SAT) is the optimization version of SAT. It has the same formulation as SAT (or *k*-SAT), but the task is to maximize the number of satisfied clauses. It is harder than SAT as one cannot guarantee in polynomial time the optimality of the solution (unlike for SAT), for problems that do not admit full satisfiability. Thus, although MaxSAT is NP-Hard, it is not known to be in NP unless P = NP. Several discrete algorithms were developed for MaxSAT, including statistical mechanics inspired methods such as Survey Propagation^37,38^. SAT and MaxSAT have a very large number of applications, with SAT solvers becoming an important back-end technology. Applications include scheduling, planning and automated reasoning, electronic design automation, bounded model checking, design of experiments, coding theory, cryptography, and drug design, see ref. ^39–41^.

A continuous-time deterministic system (CTDS) based on ordinary differential equations (ODEs), was recently proposed as an analog SAT solver, in ref. ^42^. It was designed such that all the SAT solutions appear as attractive fixed points for the dynamics while no other attractors exist trapping the dynamics. For hard problems its behavior becomes chaotic, showing that problem hardness and chaos^43,44^ are related notions within this context, and thus chaos theory can be used to study computational complexity. For fully satisfiable SAT problems the chaos is necessarily transient^45^, with the trajectory eventually settling onto one of its attracting fixed points (a SAT solution). Using numerical experiments, the CTDS was shown to solve hard SAT problems in polynomial continuous-time^42^, but at the expense of auxiliary variables growing exponentially. In a hardware realization, this implies a trade-off between time and energy costs. However, since one can control/generate energy much better than time itself, this presents a viable option for time-critical applications.^46^ proposed an analog circuit design for the CTDS, showing a 10^4^-fold speedup on hard 3-SAT problems, when compared to state-of-the-art SAT solvers^47,48^ on digital machines.

Here, we present an extension of the CTDS such as to solve MaxSAT problems. The idea is based on the observation that the CTDS makes no assumptions about problem satisfiability and thus, even for unsatisfiable SAT problems, the dynamics will still minimize the number of unsatisfied clauses. What we need to determine, however, is the likelihood of the optimality of the best solution found by analog time *t*, as function of *t*, which we achieve heuristically, by analyzing the statistics of a dynamical invariant, the escape rate^45^. In the following we will refer to our analog MaxSAT solver as Max-CTDS. We test its performance using hard benchmark MaxSAT problems, in particular, on all the 454 random benchmark problems from the 2016 MaxSAT competition^49^, showing that it achieves the same or very close results to the overall best competition solver. As another application of our approach, we consider the famous problem of Ramsey numbers^50,51^. The Ramsey number *R*(*m*, *m*) is the smallest order of a complete graph such that any coloring of its edges with two colors has a monochromatic clique of order *m*. The SAT formulation of this problem, of finding a coloring without monochromatic *m*-cliques, is fully satisfiable below the Ramsey number, whereas at the Ramsey number, it becomes MaxSAT for the first time. *R*(5, 5) is still open, only the bounds 43 ≤ *R*(5, 5) ≤ 48 are known^52,53^. Finding Ramsey numbers is challenging due to the convoluted structure of the search space, and its sheer size: there are $2^{{\left({{N}\atop {2}}\right)}}$ possible colorings of a complete labeled graph on *N* nodes (≈10^271^ for *N* = 43). For *m* = 5 (equivalent to a 10-SAT/MaxSAT problem) Max-CTDS finds good colorings for up to *N* = 42, whereas for *N* = 43 finds a coloring with only two monochromatic 5-cliques sitting on 6 nodes, the lowest energy coloring found so far, to our best knowledge, adding further support to the conjecture that *R*(5, 5) = 43. We conclude with a discussion on analog solvers and their realization in hardware.

## Results

### A continuous-time dynamical system solver for MaxSAT

Here, we focus on continuous-time systems, in which both the state variables **s** = (*s*_1_, …, *s*_*N*_) and the time variable *t* are real numbers, $s_i \in {\Bbb R}$, $t \in {\Bbb R}$, updated continuously by the algorithm (“software”), in form of a set of ordinary differential equations (ODEs)^54^ *d***s**/*dt* = **F**(**s**(*t*), *t*), $t \in {\Bbb R}$, see ref. ^32^ for a review. The process of computation is interpreted as the evolution of the trajectory (the solution to the ODEs) **s**(*t*) = **Ψ**_*t*_(**s**_0_), toward an attractive fixed-point state s^*^: lim_*t*→∞_ **Ψ**_*t*_(**s**_0_) = s^*^, representing the answer/solution to the problem. Clearly, we want to find s^*^, and the challenge is to design **F** such that the solutions to the problem (when they exist) appear as attractive fixed points for the dynamics and no other, nonsolution attractors exist that could trap the dynamics.

Our MaxSAT solver is based on a previously introduced SAT solver^42^, which we now briefly describe; more details are given in Methods. Let us assign the variable *s*_*i*_ = 2*x*_*i*_ − 1 to every Boolean variable *x*_*i*_ (when *x*_*i*_ = 0, *s*_*i*_ = −1 and when *x*_*i*_ = 1, *s*_*i*_ = 1), but allow *s*_*i*_ to vary continuously in the [−1, 1] interval. The continuous dynamical system ${\textstyle{{d{\bf{s}}} \over {dt}}} = {\dot{\mathrm s}} = {\bf{F}}$ thus generates a trajectory confined to the hypercube ${\cal H}_N$ = [−1, 1]^*N*^ with the SAT solutions s^*^ all located in its corners. To every clause *C*_*m*_ (constraint) we associate the analog clause function $K_m({\bf{s}})$ = $2^{ - k}\mathop {\prod}\nolimits_{j = 1}^N \left( {1 - c_{mj}s_j} \right)$, where *c*_*mj*_ = 1 (−1) if variable *x*_*i*_ appears in normal (negated) form in clause *C*_*m*_, and *c*_*mj*_ = 0 if it is missing (in either form) from *C*_*m*_. The normalization 2^−*k*^ ensures that *K*_*m*_ ∈ [0, 1]. One can easily check that *K*_*m*_ = 0 in a corner **s** of ${\cal H}_N$ if and only if (iff) clause *C*_*m*_ is satisfied at **s**. We then introduce a “potential energy” function *V* that depends on the *K*_*m*_-s such that *V* = 0 iff all the clauses are satisfied, that is, *K*_*m*_ = 0, ∀*m* = 1, …, *M*:

1 $$V({\bf{s}},{\bf{a}}) = \mathop {\sum}\limits_{m = 1}^M {\kern 1pt} a_mK_m({\bf{s}})^2.$$

Here, the *a*_*m*_ are time-dependent, positive weights, or auxiliary variables, *a*_*m*_(*t*) > 0, ∀*m* = 1, …, *M*, ∀*t* ≥ 0. If they were constants, the dynamics would easily get stuck in nonsolution attractors. To prevent that, the dynamics of the auxiliary variables *a*_*m*_ is coupled with the evolution of the clause functions *K*_*m*_. The dynamics of the full system is defined via

2 $$\left\{ \begin{array}{l}\dot s_i = \frac{{ds_i}}{{dt}} = - \left( {\nabla _sV} \right)_i = - \frac{\partial }{{\partial s_i}}V({\bf{a}},{\bf{s}}) = \mathop {\sum}\limits_{m = 1}^M {2a_mc_{mi}K_{mi}K_m,i = 1, \ldots ,N} \\ {\hskip -11.3pc} \dot a_m = \frac{{da_m}}{{dt}} = a_mK_m,m = 1, \ldots ,M,\end{array} \right.$$

where $K_{mi}$ = $2^{ - k}\mathop {\prod}\nolimits_{j = 1,j \ne i}^N \left( {1 - c_{mj}s_j} \right)$. Note that (2) is just a gradient descent in **s**-space on *V*, ${\dot{\mathrm s}}$ = −∇_*s*_*V*. For hard (but satisfiable) SAT formulas the dynamics is transiently chaotic, but eventually all trajectories converge to a solution. Since the dynamics is hyperbolic^42^, the probability *p*(*t*) of a trajectory no*t* finding a solution by analog time *t* decreases exponentially: *p*(*t*) ~ *e*^−*κt*^. The decay rate *κ* is an invariant of transient chaos, called the escape rate^55,56^, and it characterizes the hardness of the given SAT formula/instance.

**Fig. 1:**
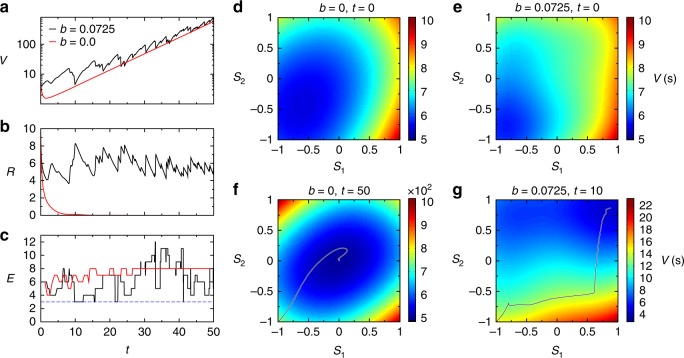
MaxSAT solver dynamics. The Max 3-SAT formula used here has *N* = 10, *M* = 80 (clauses given in the Supplementary Data 1). **a** The potential *V*, **b** the radius $R = \sqrt {\mathop {\sum}\nolimits_i {\kern 1pt} s_i^2}$, and **c** the number of unsatisfied clauses (energy) *E* as function of analog time *t* for the original dynamics corresponding to *b* = 0 (red) and the modified dynamics with *b* = 0.0725 (black). **d**–**g** Colormaps of the potential *V*(**s**(*t*), **a**(*t*)) in the plane (*s*_1_, *s*_2_). At a given time instant *t* we fix all values *s*_*j*_(*t*), *j* = 3, …, *N* and *a*_*m*_(*t*), ∀*m* and change only *s*_1_, *s*_2_ in the [−1, 1] × [−1, 1] plane, showing the instantaneous potential energy landscape *V* in this plane. The curves indicate the projection of the trajectory onto (*s*_1_, *s*_2_) up to the indicated time *t*. In *t* = 0, *s*_1_ = *s*_2_ = −1. For *b* = 0, the dynamics converges to **s** = 0, which is the centre of a deep well in the potential landscape. For *b* = 0.0725, the centre is not a minimum anymore and at time *t* = 10 the orthant with minimal energy *E*_min_ = 3 is found (the solution), shown as a blue dotted line in the *E*(*t*) figure

Next, we introduce a modified version of the above solver to solve MaxSAT. Note that if the global optimum s^*^ is not a solution with *V* = 0 (a true MaxSAT problem), then *V* will keep changing in time as function of the auxiliary variables. The dynamics is still biased to flow toward the orthants of ${\cal H}_N$ with low energy, and as shown, in Fig. 1a, it will find the global optimum, but it will never halt there. Naturally, the question arises: how do we know when we have hit an optimal assignment? For that we use a heuristic based on a statistical approach: we start many (relatively short) trajectories from random initial conditions, look for the lowest energy found by each trajectory and then exploit this statistic to help predict the lowest energy state and the time needed to get there by the solver.

However, Eqs. (1) and (2) cannot directly be applied to MaxSAT problems, one needs to modify the potential energy function, first. To see why, notice that the potential *V* in the center of ${\cal H}_N$, in **s** = 0, is always $V\left( {{\mathbf{0}},{\mathbf{a}}} \right)$ = $2^{ - 2k}\mathop {\sum}\nolimits_{m = 1}^M {\kern 1pt} a_m$, because *K*_*m*_(**0**) = 2^−*k*^, ∀*m*. On the other hand, in a corner **s**′ of the hypercube, where $\left| {s_i^\prime } \right| = 1$ ∀*i*, the value of each *K*_*m*_(***s***′) clause function is 0 if the clause is satisfied or 1 if it is unsatisfied, so the potential *V* in a corner is just the sum of auxiliary variables corresponding to the unsatisfied clauses, i.e., $V({\bf{s}}\prime ,{\bf{a}})$ = $\mathop {\sum}\nolimits_{\{ m:K_m \ne 0\} } {\kern 1pt} a_m$. Let *a* be the average value of the auxiliary variables in a given time instance *t*, *a* = ${\textstyle{1 \over M}}\mathop {\sum}\nolimits_{m = 1}^M {\kern 1pt} a_m$. Thus *V*(**0**, ***a***) = 2^−2*k*^ *aM* and *V*(**s**′, **a**) ≃ *aE*(**s**′), where we introduced *E*(**x**) to denote the number of unsatisfied clauses for an assignment **x**, which we will call “energy”, from here on. If **s**′ is the global optimum and it’s energy is large enough (typically at large constraint densities *α* = *M*/*N*), the center of the hypercube may have a smaller potential energy value (due to the 2^−2*k*^ factor), than any of the corners of the hypercube, and it may become a stable attractor, trapping the dynamics. Figure 1 shows an example of this trapping on a small MaxSAT problem with *N* = 10 variables and *M* = 80 clauses, given in Supplementary Data 1. To prevent this, we need to modify the potential energy function. We do this by adding a term *V*′(**s**, **a**) to *V*(**s**, **a**) such that it satisfies the following conditions: (1) it is symmetric in all *s*_*i*_ so that there is no bias introduced in the search dynamics, (2) the energy in **s** = **0** is always sufficiently large so that it never becomes an attractor, (3) the added term does not modify the energy in the corners of the hypercube, and (4) similarly to the original dynamics, **s** always stays within the hypercube ${\cal H}_N$, which demands that ∂*V*′/∂*s*_*i*_ = 0 for all *i* along the boundary of ${\cal H}_N$. We may imagine this added term in the form of a “hat” function: it has a maximum at **s** = **0** that keeps growing together with the time-dependent auxiliary variables (never to become permanently smaller than the potential energy in the global optimum), but vanishing at the boundary surface of the hypercube.

There are several possibilities for such terms, here we focus on one version that works well in simulations:

3 $$V({\bf{s}},{\bf{a}}) = \mathop {\sum}\limits_{m = 1}^M {\kern 1pt} a_mK_m({\bf{s}})^2 + b\alpha a\mathop {\sum}\limits_{i = 1}^N {\kern 1pt} {\mathrm{cos}}^2\left( {\frac{\pi }{2}s_i} \right),$$

where *a* is the average value of the auxiliary variables, *α* = *M*/*N* is the constraint density and *b* is a constant factor tuning the strength of the last term to be always larger than the first, when chosen properly. The sum with the ${\mathrm{cos}}^2\left( {\pi s_i{\mathrm{/}}2} \right)$ terms ensures the symmetric hat form, vanishing in the corners of ${\cal H}_N$. Note that the first term on the rhs of (3) is never larger than *aM*. We now have *V*(**0**, **a**) = (2^−2*k*^ + *b*)*aM* and *b* can be chosen such as to avoid the trapping phenomenon by the origin as described above, see also Fig. 1b. To do that, we simply demand that the potential in the origin *V*(**0**, **a**) keeps growing approximately at the same rate as the potentials in the corners of the hypercube, never getting smaller than the potential in the global minimum (the smallest potential value in the corners). Thus, as long as *V*(**0**, **a**) ≥ *V*(**s**′, **a**), where **s**′ is some corner of the hypercube accessed by the dynamics, the dynamics will not get attracted by the origin of the hypercube. Since *V*(**s**′, **a**) ≃ *aE*(**s**′), this implies that $b \ge {\textstyle{1 \over M}}E({\bf{s}}\prime ) - 2^{ - 2k}$, where *E*(**s**′)/*M* is the fraction of unsatisfied clauses in **s**′. Clearly, the *b* value can be chosen arbitrarily large, however, if it is too large, then it forces the dynamics to keep running close to the boundary of the hypercube, somewhat lowering its performance. In practice, an *E*′ = *E*(**s**′) is easily found by running a trajectory with a sufficiently large *b* value for some short time, then resetting $b \ge {\textstyle{{E\prime } \over M}} - 2^{ - 2k}$. If chosen this way, the search dynamics would not be too sensitive to this parameter *b*. The new dynamical system is therefore:

4 $$\left\{ \begin{array}{l}\dot s_i = - \frac{{\partial V}}{{\partial s_i}} = \mathop {\sum}\limits_{m = 1}^M {2a_mc_{mi}K_{mi}({\bf{s}})K_m({\bf{s}}) + \frac{\pi }{2}b\alpha a\,{\mathrm{sin}}\left( {\pi s_i} \right),\forall i = 1, \ldots ,N} \\ {\hskip -12.8pc} \dot a_m = a_mK_m,\forall m = 1, \ldots ,m.\end{array} \right.$$

Figure 1 illustrates the difference between the two dynamics (see also Supplementary Fig. 1). While for *b* = 0 (original system) the dynamics converges rapidly to **s** = **0** (seen, e.g., by monitoring the radius $R^2 = \mathop {\sum}\nolimits_i {\kern 1pt} s_i^2 \to 0$), the modified system with a properly chosen *b* > 0 continues the search. It finds an orthant with the minimum energy quite quickly (by *t* = 10), but it does not halt there, it continues the dynamics and returns to this minimum repeatedly (e.g., around *t* ≈ 16, 22, 41). Figure 1d–g shows the potential energy function landscape *V*(**s**, **a**) in the (*s*_1_, *s*_2_) plane.

### An energy-dependent escape rate

**Fig. 2:**
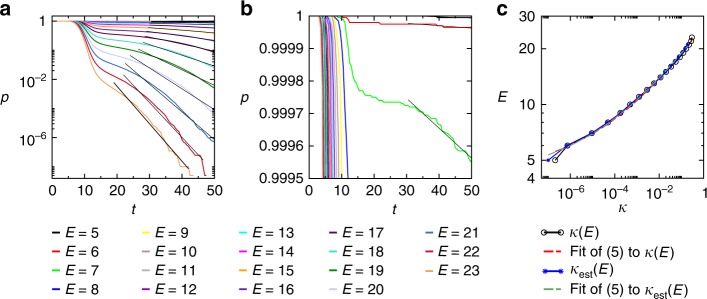
Energy dependent escape rate. **a** The *p*(*E*, *t*) distributions for a hard, benchmark MaxSAT competition problem with *N* = 250, *M* = 1000 (*α* = 4.0), “HG-3SAT-V250-C1000-1.cnf”, from ref. ^49^. We find *E*_min_ = 5 with our algorithm after running Γ = 2 × 10^5^ trajectories with *b* = 0.002375. The escape rates are obtained from fitting *p* ~ *e*^−*κt*^ onto the last section of the distributions (black lines), see Methods. **b** A zoom into the upper section of (**a**). **c** *E* vs. the escape rate *κ* using the values obtained from the fitting shown in (**a**) (black) and using the rough estimate for the escape rates *κ*_est_(*E*) ≃ −ln(*p*(*E*, *t*_max_))/*t*_max_ (blue). This estimation is convenient, as it is easier to automate in the algorithm than the fitting procedure (see Methods). The dashed lines show the fitting of Eq. (5) to the *κ*(*E*) points from (**a**) (red curve: *E* = 4.25 + 22.87*κ*^0.184^) and to the *κ*_est_(*E*) points (green curve: *E* = 4.06 + 22.31*κ*^0.178^), respectively. Both curves result in *E*_0_ ∈ (4, 5] thus predicting the global optimum $E_{{\mathrm{min}}}^{{\mathrm{pred}}} = 5$

The escape rate is an invariant measure of the dynamics introduced for characterizing transiently chaotic systems^55,56^. In a transiently chaotic system the asymptotic dynamics is not chaotic, but, for example, settles onto a simple attractor, or escapes to infinity (in open systems), however, the nonasymptotic dynamics is chaotic, usually governed by a chaotic repeller. It is well known that for hyperbolic, transiently chaotic dynamical systems the probability of a randomly started trajectory not converging to an attractor by time *t* (i.e., not finding a SAT solution in our case) decreases exponentially in time: *p*(*t*) ~ *e*^−*κt*^, where *κ* is the escape rate^45,55^. The escape rate can also be interpreted as the inverse of the average lifetime *τ* of trajectories *κ* = 1/*τ*. For permanently chaotic systems, such as our MaxSAT solver, however, this definition does not work, as there is no simple asymptotic attractor in the dynamics and the system is closed. To be able to use a similar notion also for MaxSAT, we use a thresholding on the energy of the visited states. More precisely, we monitor the probability *p*(*E*, *t*) that a trajectory has not yet found an orthant of energy smaller than *E* by analog time *t*. Here, *E* acts as a parameter of the distribution. This can be measured by starting many trajectories from random initial conditions and monitoring the fraction of those that have not yet found a state with an energy less than *E* by analog time *t*. In Fig. 2a we show these distributions for different *E* values for a MaxSAT problem. For large *E*, all trajectories almost immediately find orthants with fewer unsatisfied clauses, but for lower *E* values the distributions decay exponentially. We call their decay rates energy-dependent escape rates *κ*(*E*). Naturally, if an energy level does not exist in the system (e.g., for *E* < *E*_min_), the escape rate for that energy level is meaningless (extrapolates to zero or a negative number). This suggests that the *κ*(*E*) dependence could be used to predict where this minimum energy is reached. However, to capture this energy limit, it is more convenient to plot the *E*(*κ*) function, instead (see Fig. 2b). From extensive simulations, we observe a power-law behavior with an intercept *E*_0_:

5 $$E = E_0 + c\kappa ^\beta .$$

Since *E*_0_ is not an integer in general, we have $E_{{\mathrm{min}}} = \left\lfloor {E_0} \right\rfloor + 1$. This observation is at the basis of our method to predict the global energy minimum for MaxSAT.

### Procedure for predicting the global minimum

Here, we describe the algorithm along with the halting criterion for system (4) with details presented in the Methods section along with a flowchart shown in Supplementary Fig. 2. The exponentially decaying nature of the *p*(*E*, *t*) distributions implies that sooner or later every trajectory will visit the orthant with the lowest energy. Nevertheless, instead of leaving one trajectory to run for a very long time, it is more efficient starting many shorter trajectories from random initial conditions and tracking the lowest energy reached by each trajectory (see Supplementary Fig. 3). This also generates good statistics for *p*(*E*, *t*) and for obtaining the properties of the chaotic dynamics that are then exploited along with (5) to predict the value of the global minimum and to decide on the additional number of trajectories needed to find a lower energy state with high probability.

The basic step of the algorithm is to run a trajectory *ω* from a random initial condition up to a given time *t*_max_ and record the lowest energy found by this particular trajectory, denoted by *E*_*s*_(*ω*). Let Γ denote the total number of trajectories run so far, ${\cal T}$ the set of these trajectories (thus Γ = $\left| {\cal T} \right|$), and $\overline E ({\mathrm{\Gamma }})$ = ${\mathrm{min}}_{\omega \in {\cal T}}{\kern 1pt} E_s(\omega )$ be the lowest energy found by all these trajectories. Using statistical methods and the relation between energy and escape rate *κ*(*E*) (shown in (5)), the algorithm repeatedly predicts (as Γ grows) the expected number of trajectories we need to run in total to find the lower energy value $\overline E - 1$, i.e., ${\mathrm{\Gamma }}^{{\mathrm{pred}}}\left( {\overline E - 1} \right)$ and the global minimum energy $E_{{\mathrm{min}}}^{{\mathrm{pred}}}$. We then monitor $E_{{\mathrm{min}}}^{{\mathrm{pred}}}$ for saturation and once the saturation criterion is reached, it outputs a decision $E_{{\mathrm{min}}}^{{\mathrm{dec}}}$, representing the final energy value predicted by the algorithm as the global minimum. If this energy value has already been attained (found at least one assignment for it), the algorithm outputs the corresponding assignment(s). If it did not attain it then it keeps running until finds such an assignment or reaches the preset maximum limit Γ_max_ on the number of runs. In the latter case it outputs the lowest energy value attained and the corresponding assignment(s) and the consistency status of the predicted value.

### Performance on random Max 3-SAT problems

**Fig. 3:**
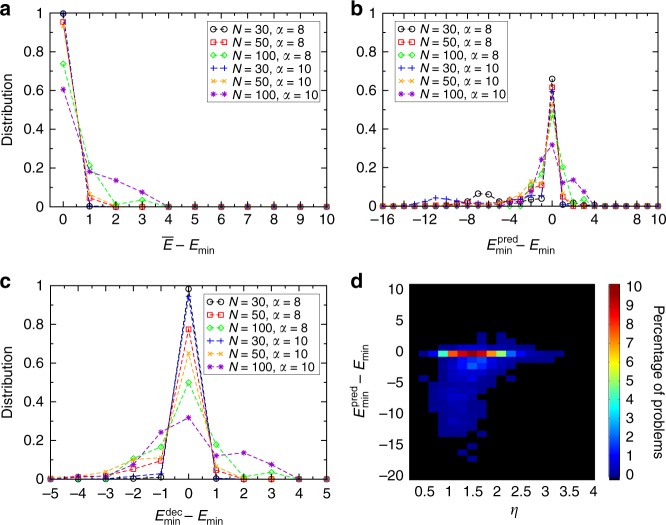
Algorithm statistics over random Max 3-SAT problems. Distribution of differences between the real global minimum *E*_min_ obtained with the exact algorithm MaxSatz and **a** the smallest energy found by the algorithm $\overline E$, **b** the predicted minimum value $E_{{\mathrm{min}}}^{{\mathrm{pred}}}$, and **c** the final decision of the algorithm $E_{{\mathrm{min}}}^{{\mathrm{dec}}}$ shown for problems with different *N* and *α* values (see legends). **d** The percentage of instances indicated by color (see color bar) for different values of the error $E_{{\mathrm{min}}}^{{\mathrm{pred}}} - E_{{\mathrm{min}}}$ and hardness *η*. Most instances are in the $E_{{\mathrm{min}}}^{{\mathrm{pred}}} - E_{{\mathrm{min}}} = 0$ row indicating correct prediction. Large errors occur mainly at smaller *η* values, and are dominantly negative

We first test our algorithm and its prediction power on a large set (in total 4000) of random Max 3-SAT problems with *N* = 30, 50, 100 variables and constraint densities *α* = 8, 10. (In 3-SAT the SAT-UNSAT transition is around *α* ≃ 4.267^57^). We compare our results with the true minimum values (*E*_min_) provided by the exact algorithm MaxSATZ^58,59^. In Fig. 3, we compare the lowest energy found by the algorithm $\overline E$, the predicted minimum $E_{{\mathrm{min}}}^{{\mathrm{pred}}}$ and the final decision by the algorithm $E_{{\mathrm{min}}}^{{\mathrm{dec}}}$ with the true optimum *E*_min_, by showing the distribution of their deviations from *E*_min_ across many random problem instances. We use *t*_max_ = 25 and at most Γ_max_ = 150,000 runs, after which we stop the algorithm even if the prediction is not final. Thus, one expects that the performance of the algorithm decreases as *N* increases, (e.g., at *N* = 100), so that we would need to run more trajectories to obtain the same performance. Nevertheless, the results show that all three distributions have a large peak at 0. Most errors occur in the prediction phase, but many of these can be significantly reduced through simple decision rules (see Methods), because they occur most of the time at easy/small problems, where the statistics is insufficient (e.g., too few points since there are only few energy values). To show how the error in prediction depends on the hardness of problems, we studied the correlation between the error $E_{{\mathrm{min}}}^{{\mathrm{pred}}} - E_{{\mathrm{min}}}$ and the hardness measure applicable to individual instances *η* = −ln *κ*/ln *N* (see ref. ^43^), see Fig. 3d (and Supplementary Fig. 4). Interestingly, larger errors occur mainly at the easiest problems with *η* < 2. Calculating the Pearson correlation coefficient between $\left| {E_{{\mathrm{min}}}^{{\mathrm{pred}}} - E_{{\mathrm{min}}}} \right|$ and *η* (excluding instances where the prediction is correct) we obtain a clear indication that often smaller *η* (thus for easier problems) generates larger errors. Positive errors are much smaller and shifted toward harder problems. Negative errors mean that the algorithm consistently predicts a slightly lower energy value than the optimum, which is good as this gives an increased assurance that we have found the optimum state. In Supplementary Fig. 4b, we show the correlation coefficients calculated separately for problems with different *N* and *α*.

### Performance evaluation on hard MaxSAT competition problems

Next, we present the performance of our solver on MaxSAT competition problems, from 2016^49^. We are foremost interested if Max-CTDS is capable of predicting the global minimum energy value and finding assignments corresponding to that energy value for hard problems, within a reasonable time.

**Fig. 4:**
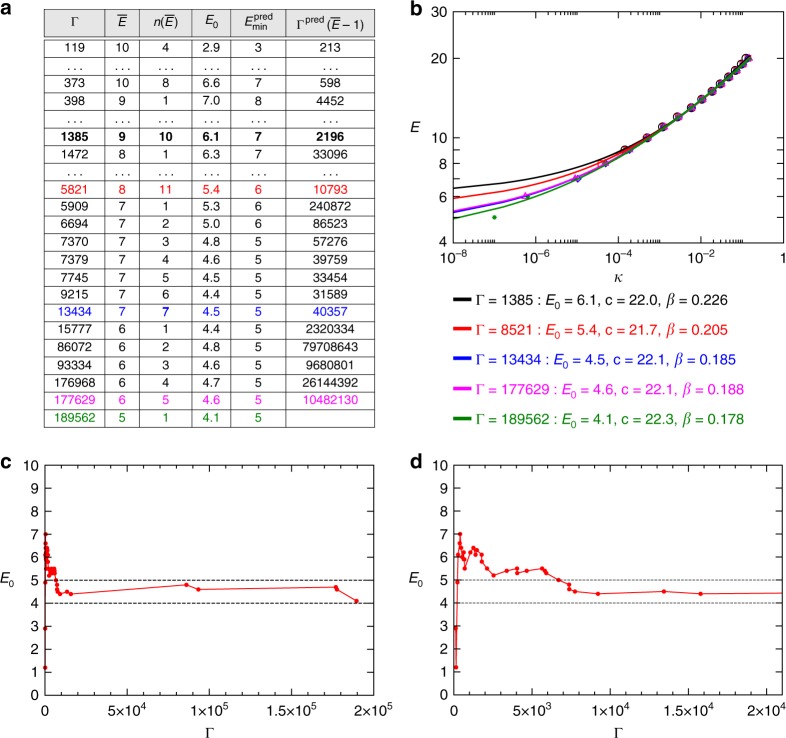
Algorithm performance on a hard benchmark problem. We use the same problem as in Fig. 2. **a** Γ is the number of trajectories, $\overline E$ the lowest energy found until that point, $n\left( {\overline E } \right)$ is the number of times this energy has been found, *E*_0_ is the parameter obtained from fitting of Eq. (5), $E_{{\mathrm{min}}}^{{\mathrm{pred}}}$ and estimating ${\mathrm{\Gamma }}^{{\mathrm{pred}}}\left( {\overline E - 1} \right)$. The algorithm estimates the escape rate and performs a prediction at each Γ shown in the table and for the colored lines we show the fitting curves in (**b**). **c** The relevant parameter *E*_0_ is shown as function of Γ. While it wildly fluctuates at the beginning when the statistics is small, it remains in the *E*_0_ ∈ [4, 5) interval, convincingly predicting $E_{{\mathrm{min}}}^{{\mathrm{pred}}} = 5$ already after Γ = 7000 up until the point that it finds this energy at Γ = 189,562. At this point it could be expected that we do not have a good estimate for *κ*(5) because it has been found only once (*n*(5) = 1), nevertheless the estimation $E_{{\mathrm{min}}}^{{\mathrm{pred}}}$ remains consistently the same, convincing our algorithm to accept $E_{{\mathrm{min}}}^{{\mathrm{dec}}} = 5$ and stop. **d** A zoom into the [0, 2 × 10^4^] interval of (**c**)

For illustration purposes, here we first discuss an extremely hard competition problem instance with *N* = 250 variables and *M* = 1000 clauses, called “HG-3SAT-V250-C1000-1.cnf”, which was reposted for several years. This problem was also used in Fig. 2. No complete competition algorithm could solve this problem. The best complete solver in 2016, the CCLS2akms has found a state with energy value 5, but could not prove that it is optimal within the allotted time (30 min). We ran our algorithm on a regular 2012 iMac 21.5, 3.1 GHz, Intel Core i7 computer and it predicted the lowest energy of 5 (unsatisfied clauses), after 21 min 24 s of running time and produced an assignment for it after 9.168 h of running time. The minimum energy prediction was achieved already after Γ = 7000 trajectories, whereas finding an assignment with this minimum energy took a total of Γ = 189,562 trajectories to run. The minimum energy assignment corresponding to $E_{{\mathrm{min}}}^{{\mathrm{dec}}} = 5$ is provided in Supplementary Data 2. (The problem can be downloaded from the competition site^49^). We ran the complete and exact algorithm, MaxSatz^58,59^ for over 5 weeks on this problem and the smallest energy it found was *E* = 9. The details of how the Max-CTDS algorithm performs are shown in Fig. 4. Similar figures for other hard problems such as for a 4-SAT problem and a spin-glass problem are shown in Supplementary Figs. 5 and 6.

**Fig. 5:**
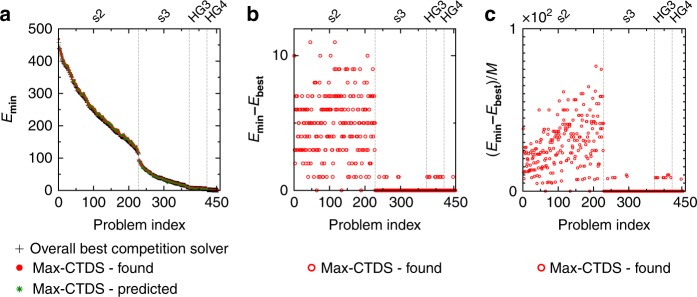
Algorithm performance on competition MaxSAT problems. **a** Using the 454 random benchmark problems from the 2016 SAT competition^49^, we compared the overall best (lowest) energy found by the competition solvers (black + symbols) and the minimum found/predicted by our Max-CTDS. There are four categories of problems, separated by vertical dashed lines: “s2” (Abrame-Habet) are Max 2-SAT with *N* ∈ [100, 200], *M* ∈ [1200, 2600]; “s3” (Abrame-Habet) are Max 3-SAT with *N* ∈ [70, 110], *M* ∈ [700,1500]; “HG3” (high-girth) are Max 3-SAT problems with *N* ∈ [250, 300], *M* ∈ [1000, 1200], and “HG4” are Max 4-SAT problems with *N* ∈ [100, 150], *M* ∈ [900, 1350]. **b** There are some small energy differences especially in the “s2” category, where the ODEs are more stiff and the integration becomes very slow. **c** The energy differences normalized by the number of clauses, (*E*_min_ − *E*_best_)/*M*. Max-CTDS solves all problems within 0.8% of the best energy value

Figure 5a shows the lowest energy values (+ symbol) for all the 454 random MaxSAT problems of the 2016 competition^49^, obtained by the (incomplete/heuristic) competition solvers, and the predicted and found lowest energies attained by Max-CTDS (green star and solid red circle symbol, respectively). See Supplementary Fig. 7 for more comparisons with other competition solvers. One can see that Max-CTDS is capable of predicting and achieving the same energy levels as the overall best, for almost all of the high-girth MaxSAT problems “HG3”, “HG4”, and “s3” (Abrame-Habet). There are, however, small deviations in the “s2” group, where Max-CTDS achieves energy levels close to the overall best. These deviations are presented in detail in Fig. 5b, while in Fig. 5c we are showing them normalized by the total number of clauses *M*, for every problem, indicating that Max-CTDS solved all the problems within 0.8% of the best value. The main reason for the discrepancy in “s2” is the stiffness of the equations^60^, in this category. Since in Max 2-SAT there are only two variables that can satisfy a clause, the problems are more constrained, and additionally, the problems in the “s2” category also have a high minimum energy value (“s2” had also the largest problems with *N* ∈ [120, 200], *M* ∈ [1200, 2600]). This causes the effects of stiffness to appear earlier in the simulations than for the other problems, slowing them down; see Supplementary Note 1 and Supplementary Fig. 8 for a more detailed description. The fact that the Max-CTDS solver still finds very close solutions even for these problems shows, that it has a “smart” search dynamics.

In terms of computation time (wall-clock time) on digital machines (using standard computers), Max-CTDS typically took on the order of hours to find an assignment for the minimum energy value. The average time over all the problems was 4.35 h, with a standard deviation of 5.05 h: in the “s2” category the average time was 10.93h, in “s3”: 0.52h, in “HG3”: 5.69h, and in “HG4”: 0.28 h. The lowest search time was 1.08s, for problem “s3v70c700-5.cnf”, with the best heuristic competition solver taking 0.64 seconds for this problem. The worst search time by Max-CTDS was for a very stiff problem, “s2v120c2500-2.cnf” at 4 days and 14.47h, with the best heuristic solver for this problem taking only 1.36s. These numbers certainly depend on the digital hardware used. Note that in an analog circuit implementation, the current flow or voltage behavior would correspond to the equations of the solver, eliminating numerical integration issues and thus the algorithm should run much faster (^46^ shows a possible 10^4^ speedup).

### Application to Ramsey numbers

Ramsey theory deals with the unavoidable appearance of order in large sets of objects partitioned into few classes, with deep implications in many areas of mathematics^51,61^ but also with practical applications^62^. Although it has several variants, in the standard, two-color Ramsey number problem we have to find the order for the smallest complete graph for which no matter how we color its edges with two colors (red and blue), we cannot avoid creating a monochromatic *m*-clique. The number of nodes for the smallest such complete graph is denoted by *R*(*m*, *m*). The proof that *R*(3, 3) = 6 is trivial. For *m* = 4 the answer is *R*(4, 4) = 18 and it is harder to prove^63^. The *m* = 5 case is still open, only the bounds 43 ≤ *R*(5, 5) ≤ 48 are known^52^. The best lower bound of 43 was first found in 1989 by Exoo^64^, and the upper bound was only recently reduced from 49^53^ to 48 by Angeltveit and McKay^65^. Using various heuristic methods, researchers have found in total 656 solutions (328 graphs and their complements) for the complete graph on 42 nodes^53^. It has been conjectured by McKay and Radziszowski^53^ that there are no other solutions for *N* = 42. Starting from these solutions they searched for a 5-clique-free coloring in 43. As no solution was found, McKay, Radziszowski, and Exoo make the strong conjecture that *R*(5, 5) = 43^53^.

To tackle Ramsey number problems with our algorithm, we first transform them into *k*-SAT^66,67^: every edge *i* (*i* = 1, …, *N*(*N* − 1)/2) to be colored is represented by a Boolean variable *x*_*i*_ (with *x*_*i*_ ∈ {0, 1}, 1 = blue, 0 = red). A clique of size *m* has *m*(*m* − 1)/2 edges. We are satisfied with a coloring (a solution) when no *m*-clique is monochromatic, i.e., every *m*-clique with set of edges {*i*_1_, …, *i*_*m*(*m*−1)/2_} must have both colors, expressed as the statement formed by the conjunction of the two clauses

6 $$\left( {x_{i_1} \vee \ldots \vee x_{i_{m(m - 1)/2}}} \right) \wedge \left( {\overline x _{i_1} \vee \ldots \vee \overline x _{i_{m(m - 1)/2}}} \right)$$

being true. This means that for every *m*-clique we have two clauses and thus there are a total of $2({{N}\atop{M}})$ clauses to satisfy. Since the number of clauses (*O*(*N*^*m*^)) for *m* ≥ 2 grows faster in *N* than the number of variables *N*, there will be a lowest *N* value corresponding to UNSAT, which is the sought *R*(*m*, *m*) Ramsey number. Thus, for *m* = 3 we have a 3-SAT problem, for *m* = 4 a 6-SAT problem and for *m* = 5 a 10-SAT problem. For graphs with *N* = 42 nodes the number of clauses is $2 \times ({{42}\atop{5}})$ = 1,701,336 and the search space has $2^{({{42}\atop{2}})}$ = 2^861^ ≃ 1.5 × 10^259^ colorings. If we were to compute the familiar constraint density *α*, it would be *α* = $2 ({{42}\atop{5}}){\mathrm{/}}({{42}\atop{2}})$ = 1976, indeed above the SAT/UNSAT transition point for random 10-SAT, which is estimated to be *α*_*s*_|_10−SAT_ ≃ 707^68^.

**Fig. 6:**
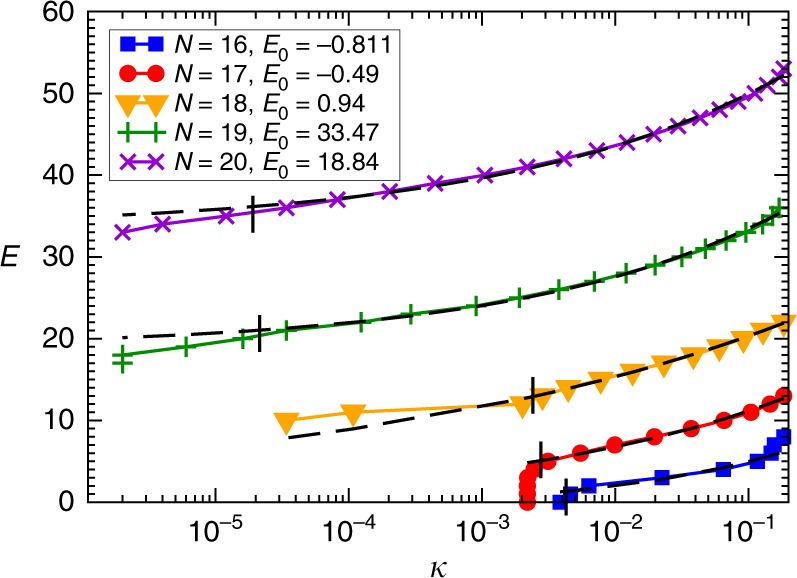
Finding the Ramsey number *R*(4, 4). The *E*(*κ*) relationship for the 6-SAT problems corresponding to the *K*_*N*_ complete graph colorings with two colors. *E*_0_ is the extrapolated value based on the fit from Eq. (5) (dashed lines). The long vertical bars indicate the lower end of the fitting range. Note that for *N* = 16, 17, *E*_0_ is a negative value indicating full colorability (the corresponding 6-SAT problem is fully satisfiable), whereas for *N* ≥ 18, *E*_0_ > 0, and thus the 6-SAT problem becomes MaxSAT

Applying our algorithm for the *m* = 4 Ramsey problem, we can easily find coloring solutions for *N* ≤ 17, while for *N* = 18 it predicts that there is no solution, indeed confirming that *R*(4, 4) = 18. This is seen from the plot of *E* vs. *κ* in Fig. 6. For *N* ≤ 17 the smooth portion of the curve fitted by (5) suddenly cuts off, *κ* being the same for all energy values lower than a threshold value, meaning that after reaching a state corresponding to the threshold energy level, the solution (i.e., *E* = 0) is immediately found. This is simply due to the fact that (5) is a statistical average behavior characteristic of the chaotic trajectory, from the neighborhood of the chaotic repeller of the dynamics and away from the region in which the solution resides. However, once the trajectory enters the basin of attraction and nears the solution, the dynamics becomes simple, nonchaotic, and runs into the solution, reflected by the sudden drop in energy. This is not due to statistical errors, because the curve remains consistent when plotting it using 10^3^, 10^4^, or 10^5^ initial conditions (the figure shows 10^5^ initial conditions).

**Fig. 7:**
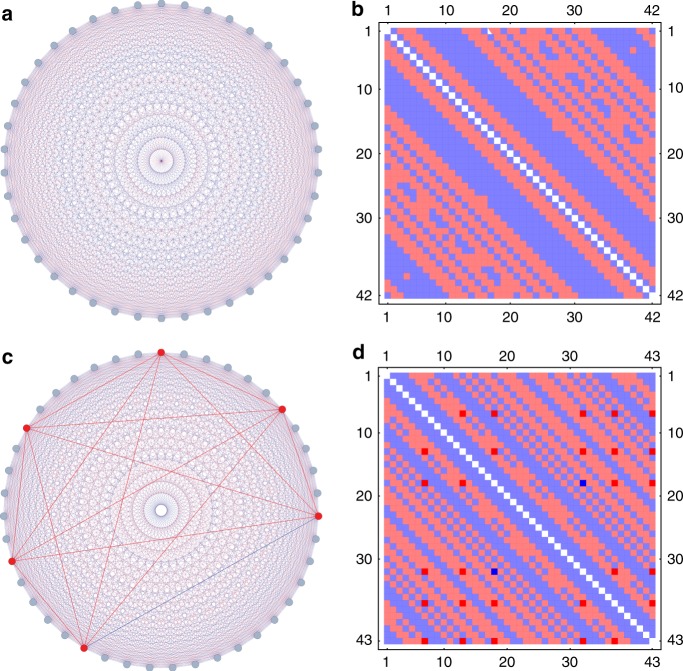
Colorings for the *R*(5, 5) Ramsey number problem. **a** A coloring of the complete graph on *N* = 42 nodes that avoids monochromatic 5-cliques. **b** The adjacency matrix corresponding to the coloring in (**a**), using the same colors. **c** The best coloring of the complete graph on *N* = 43 nodes containing only 2 monochromatic (red) 5-cliques, sitting on 6 nodes (highlighted with thicker edges). **d** The adjacency matrix corresponding to the coloring in (**c**), using the same colors. The thicker red (blue) edges from (**c**) are represented with darker red (blue) cells. Supplementary Fig. 9 shows a reordered version of this matrix such that the 5-cliques can be seen in the upper left corner of the matrix

Searching for the value of *R*(5, 5) one can relatively easily find coloring solutions without 5-cliques up to *N* = 35 for which the number of variables is 595 and the number of clauses 649,264, already huge for a 10-SAT problem for other types of SAT and MaxSAT solvers. To find solutions faster for *N* ≥ 36, however, we employ a strategy based on circulant matrices^69^ helping us find solutions (proper colorings) up to and including *N* = 42 in a relatively short time (on the order of hours), see the description in the Methods. This approach places the trajectories relatively close to the solution and a proper coloring can be found in hours even for *N* = 42, (see Fig. 7a, b, and Supplementary Data 3 for an easily readable list of edge colorings), for which other heuristic algorithms take many days of computational time^53^, even with the circulant matrix strategy. Applying the same strategy for *N* = 43 we did not find any complete coloring solutions, however, we did find a coloring that creates only two (out of $$\left( {\begin{array}{*{20}{c}} {43} \\ 5 \end{array}} \right)$$ = 962,598 possible) monochromatic 5-cliques, see Fig. 7c, d, and the specific coloring provided in Supplementary Data 4.

## Discussion

In summary, we presented a continuous-time dynamical system approach to solve a quintessential discrete optimization problem, MaxSAT. The solver is based on a deterministic set of ordinary differential equations and a heuristic method that is used to predict the likelihood that the optimal solution has been found by analog time *t*. The prediction part of the algorithm exploits the statistics of the ensemble of trajectories started from random initial conditions, by introducing the notion of energy-dependent escape rate and extrapolating this dependence to predict both the minimum energy value (lowest number of unsatisfied clauses) and the expected time needed by the algorithm to reach that value. This statistical analysis is very simple; it is quite possible that more sophisticated methods can be used to better predict minima values and time lengths. Due to its general character, the presented approach can be extended to other optimization problems as well, to be presented in forthcoming publications.

Instead of a numerical implementation on a digital computer, one would ideally like to use a direct implementation by analog circuits, the feasibility of which has been shown in ref. ^46^, as it promises to be a faster (by orders of magnitude) and more efficient approach. One reason for this is that in such analog circuits the von Neumann bottleneck is eliminated, with the circuit itself serving its own processor and memory, see ref. ^46^ for details. Implementation on a digital computer, however, (as it was done here) requires the use of an ODE integrator algorithm, which discretizes the continuous-time equations and evolves them step by step, while controlling for errors. Note that in this case the continuous time variable *t* is also simulated and evolved in steps $t \mapsto t + {\mathrm{\Delta }}t$. The time-cost of the dynamics in this case is the wall-clock time (not *t*), which also depends on the computer hardware and the numerical integration method used. However, in a physical implementation, the *t* variable would be the real time-cost of the “computation”. In digitized form, the solver is not performing better than current MaxSAT competition solvers simply because the dynamics evolves many (several thousands or more) coupled ODEs, and this integration is time consuming on digital machines. Additionally, to manage the occasional stiffness of the differential equations, one needs to use implicit or higher-order integration methods, also contributing to the slowing down of the simulations. Note that this would not be an issue for analog circuit implementations, as there are no discretization schemes or numerical integration methods; the physical system evolves its currents and voltages according to the ODEs, flowing toward a halting condition, solving the problem. Nevertheless, even when simulated on a digital machine, the solver finds very good solutions to hard problems in reasonable time. This is because continuous-time analog dynamical systems represent an entirely novel family of solvers and search dynamics, and for this reason they behave differently and thus may perform better, than existing algorithms on certain classes of hard problems.

It is also important to note that the system (4) is not unique, other ODEs can be designed with similar or even better properties. This is useful, because the form given in (4) is not readily amenable to simple hardware implementations, due to the constantly growing auxiliary variable dynamics (all variables represent a physical characteristic such as a voltage or a current and thus they will have to have an upper limit value for a given device). However, the auxiliary variables do not need to grow always exponentially, one can devise other variants in which they grow exponentially as needed, otherwise they can decay (to be presented in a future publication), allowing for better hardware implementations.

To illustrate the effectiveness of our solver, we applied it to the famous two-color Ramsey problem and in particular for *R*(5, 5), which is still open. We have shown, that the two-color Ramsey problem avoiding monochromatic *m*-cliques can be translated into an ${\textstyle{{m(m - 1)} \over 2}}$-SAT problem and thus a 10-SAT for *m* = 5. Note that digital SAT solving algorithms focus on 3-SAT or 4-SAT problems, and usually are unable to handle directly the much harder 10-SAT. Our solver when run on the corresponding 10-SAT problem was able to find colorings of the complete graph of order 42 without monochromatic 5-cliques, and a coloring with only two monochromatic 5-cliques on 6 nodes for the complete graph on 43 vertices (colorings in the literature for *N* = 43 quote 500+ monochromatic 5-cliques^70^). Note that after posting our paper to arxiv, Geoffrey Exoo in a private communication mentioned that he also found the same, smallest energy coloring as presented here. This adds further support to the conjecture that *R*(5, 5) = 43.

## Methods

### SAT/MaxSAT definitions

Boolean satisfiability in conjunctive normal form (CNF) is a constraint satisfaction problem formulated on *N* Boolean variables *x*_*i*_ ∈ {0, 1}, *i* = 1, …, *N* and *M* clauses *C*_1_, …, *C*_*M*_. A clause is the disjunction (OR operation) of a set of literals, a literal being either the normal (*x*_*i*_) or the negated (NOT) form $\left( {\overline x _i} \right)$ of a variable, an example clause being: *C*_4_ = $\left( {x_9 \vee \overline x _{10} \vee x_{27}} \right)$. The task is to find an assignment for the variables such that all clauses are satisfied, or alternatively, the conjunctive formula ${\cal F}$ = *C*_1_∧ … ∧*C*_*M*_ evaluates to 1 (TRUE). If all clauses contain exactly *k* literals, the problem is *k*-SAT. For *k* ≥ 3 this is an NP-complete decision problem^35^, meaning that a candidate solution is easily (poly-time) checked for satisfiability, but finding a solution can be hard (exp-time). Oftentimes, when studying the performance of algorithms over sets of randomly chosen problems the constraint density *α* = *M*/*N* is used as a statistical guide to problem hardness^71,72^. MaxSAT has the same formulation as SAT, but the task is to maximize the number of satisfied clauses. For both SAT and MaxSAT, all known algorithms require exponentially many computational steps (in *N*) in the worst case, to find a solution. However, guaranteeing optimality of solution for MaxSAT is as hard as finding the solution itself, and thus MaxSAT is harder than SAT (NP-hard). Max 2-SAT (i.e., *k* = 2) is already NP-hard.

### Properties of the analog SAT solver

Eq. (2) preserves the positivity of the auxiliary variables at all times, since *K*_*m*_ ≥ 0. According to (2), the auxiliary variables grow exponentially whenever the corresponding clause functions are not satisfied, however, once *K*_*m*_ = 0, $\dot a_m$ = 0, they stop growing. Eq. (2) ensures that whenever the dynamics would get stuck in a local, nonsolution minimum of *V*, the exponential acceleration changes the shape of *V* such as to eliminate that local minimum. This can be seen by first solving formally (2): *a*_*m*_(*t*) = $a_{m0}\,{\mathrm{exp}}\left( {{\int}_0^t {\kern 1pt} d\tau K_m({\bf{s}}(\tau ))} \right)$, then inserting it into (1): *V* = $\mathop {\sum}\nolimits_{m = 1}^M {\kern 1pt} a_{m0}e^{{\int}_0^t {\kern 1pt} d\tau K_m}K_m^2$. Due to the exponentially growing weights, the changes in *V* are dominated by the clause that was unsatisfied the longest. Keeping only that term in *V* and inserting it into (2), it is easily seen that the dynamics drives the corresponding clause function toward zero exponentially fast, until another clause function takes over. This is repeated until all clauses are satisfied, for solvable SAT problems. The properties and performance of this solver have been discussed in previous publications^42–44^. In^43^ we show that the notion of escape rate *κ* can be used to characterize the hardness of individual problem instances. We demonstrate this on Sudoku puzzles (all Sudoku problems can easily be translated into SAT), showing that $\eta = - {\mathrm{log}}_{10}{\kern 1pt} \kappa$ indeed provides a good hardness measure that also correlates well with human ratings of puzzle hardness.

### Algorithm description

Here, we give a simple, nonoptimized variant of the algorithm (see flowchart in Supplementary Fig. 2). Better implementations can be devised, for example with better fitting routines, however the description below is easier to follow and works well. Given a SAT problem, we first determine the *b* parameter as described previously. Step 1: initially we set $\overline E = M$, Γ_min_, ${\mathrm{\Gamma }}_{{\mathrm{max}}} \gg {\mathrm{\Gamma }}_{{\mathrm{min}}}$, ${\mathrm{\Gamma }}^{{\mathrm{pred}}}\left( {\overline E - 1} \right)$ = ${\mathrm{\Gamma }}_{{\mathrm{min}}} + 1$ and *t*_max_. Unless specified otherwise, in our simulations we used Γ_min_ = 100, Γ_max_ = 2 × 10^6^, *t*_max_ = 50. Step 2: to initialize our statistics, we run Γ_min_ trajectories up to *t*_max_, each from a random initial condition. For every such trajectory *ω* we update the *p*(*E*, *t*) distributions as function of the energies of the orthants visited by *ω*. We record the lowest energy value found $\overline E \left( {{\mathrm{\Gamma }}_{{\mathrm{min}}}} \right)$. Step 3: starting from Γ = Γ_min_ + 1 and up to Γ_max_, we continue running trajectories in the same way and for each one of them check: (a) If $E_s \le \overline E$, set $\overline E {\kern 1pt}_ {\mathrm{min}}:=\left( {E_s,\overline E } \right)$, update *p*(*E*, *t*) and go to Step 4. (b) If Γ just reached ${\mathrm{\Gamma }}^{{\mathrm{pred}}}\left( {\overline E - 1} \right)$, go to Step 4. (c) If Γ = Γ_max_, output “Maximum number of steps reached, increase Γ_max_”, output the lowest energy value found, the predicted $E_{{\mathrm{min}}}^{{\mathrm{pred}}}$ and the quality of fit for $E_{{\mathrm{min}}}^{{\mathrm{pred}}}$, then halt. Step 4: using the *p*(*E*, *t*) distributions, estimate the escape rates *κ*(*E*) as described in the corresponding Methods section. Step 5: the *κ*(*E*) curve is extrapolated to the *E* − 1 value obtaining *κ*(*E* − 1) and then using this we predict ${\mathrm{\Gamma }}^{{\mathrm{pred}}}\left( {\overline E - 1} \right)$ (as described in another Methods section). Further extrapolating the *κ*(*E*) curve to *κ* = 0 we obtain $E_{{\mathrm{min}}}^{{\mathrm{pred}}}$ (see the corresponding Methods section). Step 6: we check the consistency of the prediction defined here as saturation of the predicted values. We call it consistent, if $E_{{\mathrm{min}}}^{{\mathrm{pred}}}$ has not changed during the last 5 predictions. If it is not consistent yet, we continue running new trajectories (Step 4). If the prediction is consistent, we check for the following halting conditions: (i) If $E_{{\mathrm{min}}}^{{\mathrm{pred}}} = \overline E ({\mathrm{\Gamma }})$ then we decide the global optimum has been found: $E_{{\mathrm{min}}}^{{\mathrm{dec}}} = E_{{\mathrm{min}}}^{{\mathrm{pred}}} = \overline E ({\mathrm{\Gamma }})$ and skip to Step 7. (ii) If the fitting is consistently predicting $E_{{\mathrm{min}}}^{{\mathrm{pred}}} > \overline E ({\mathrm{\Gamma }})$ (usually it is very close, $\overline E ({\mathrm{\Gamma }}) + 1$) we check the number of trajectories that has attained states with $\overline E ({\mathrm{\Gamma }})$, i.e., $n\left( {\overline E } \right)$ = $\left[ {1 - p\left( {\overline E ({\mathrm{\Gamma }}),t_{{\mathrm{max}}}} \right)} \right]{\mathrm{\Gamma }}$. If it is large enough (e.g. >100), we decide to stop running new trajectories and set $E_{{\mathrm{min}}}^{{\mathrm{dec}}} = \overline E ({\mathrm{\Gamma }})$ and go to Step 7. (iii) If $E_{{\mathrm{min}}}^{{\mathrm{pred}}} < \overline E ({\mathrm{\Gamma }})$ then we most probably have not found the global optimum yet and we go to Step 4. We added additional stopping conditions that can shorten the algorithm in case of easy problems, see Methods corresponding section, but these are not so relevant. Step 7: the algorithm ends and outputs $E_{{\mathrm{min}}}^{{\mathrm{pred}}}$, $E_{{\mathrm{min}}}^{{\mathrm{dec}}}$, $\overline E$ values, the Boolean variables corresponding to the optimal state found, along with the quality of fit.

### Estimation of the escape rates

As seen in Fig. 2 and Supplementary Fig. 1 the exponential decay of the *p*(*E*, *t*) distribution settles in after a short transient period. Theoretically the escape rate can be obtained by fitting the exponential on that last part of the curves (Fig. 2a). However, while running the algorithm it would be difficult to automatically estimate the region where the exponential should be fitted. The simple approach that works well is to estimate the escape rates as *κ*(*E*) ≃ −ln(*p*(*E*, *t*_max_)/*t*_max_, which practically would correspond to the exponential behavior being valid on the whole (0, *t*_max_) interval. Note, the *p*(*E*, *t*) is a cumulative distribution with *p*(*E*, 0) = 1. Usually this estimation is very close to the fitted values (Fig. 2c), but notice that what matters here is the scaling behavior of the escape rates, and this is quite precisely obtained this way because it simply uses the scaling behavior of the *p*(*E*, *t*_max_) values, instead of the fittings, which is sensitive to the chosen interval.

### Predicting the number of trajectories

After calculating the escape rates *κ*(*E*) one can estimate the number of expected trajectories needed to find a lower energy value: ${\mathrm{\Gamma }}^{{\mathrm{pred}}}\left( {\overline E - 1} \right)$ as described below. Clearly, *p*(*E*, *t*_max_) is the probability that a trajectory has not reached the energy level *E* up to time *t*_max_. This means that 1 − *p*(*E*, *t*_max_) is the probability that a trajectory did reach energy *E*, up to time *t*_max_. Running Γ trajectories, thus $n\left( {\overline E } \right)$ = $\left[ {1 - p\left( {E,t_{{\mathrm{max}}}} \right)} \right]{\mathrm{\Gamma }}$ will give the expected number of trajectories that reached energy *E*. Thus, the expected number of trajectories we need to run in total to find the $\overline E - 1$ energy value at least once is:

7 $${\mathrm{\Gamma }}^{{\mathrm{pred}}}\left( {\overline E - 1} \right) = \frac{1}{{1 - p\left( {t_{{\mathrm{max}}},\overline E - 1} \right)}}.$$

However, no trajectory has reached energy $\overline E - 1$ yet, and thus we don’t have $p\left( {\overline E - 1,t_{{\mathrm{max}}}} \right)$. Instead, it is computed from $p\left( {\overline E - 1,t_{{\mathrm{max}}}} \right)$ ≃ $e^{ - \kappa \left( {\overline E - 1} \right)t_{{\mathrm{max}}}}$, after extrapolating the *κ*(*E*) curve to obtain $\kappa (\overline E - 1)$.

### Predicting the global optimum

When fitting the curve *E* = *E*_0_ + *aκ*^*β*^ on our data points we used the Numerical Recipes implementation^73^ of the Levenberg–Marquardt nonlinear curve fitting method^74,75^. This implementation has some weaknesses, one could choose to use other implementations or other methods. Sometimes a three-parameter fitting is too sensitive and does not give good results. Because we do not need a very precise value for *E*_0_ (we just need to find the integer interval it falls into, because $E_{{\mathrm{min}}}^{{\mathrm{pred}}} = [E_0] + 1$, [.] meaning the integer part) we perform a series of fittings always fixing *E*_0_ and leaving only 2 unknown parameters (*a*, *β*). For each *E*_0_ = $\overline E ,\overline E - 0.1,\overline E - 0.2, \ldots .$ we then perform a fitting and check the *χ*^2^ error. The fitting with minimal error is chosen as the final *E*_0_ and final fitted curve.

### Additional stopping conditions

There are cases usually for easy problems, when the fitting using the form (5) does not work well, but based on certain simple conditions we can trust that the global optimum has been found. For example, if there aren’t enough (e.g., less than 5) data points in the *κ*(*E*) curve fitted (this partly explains why the fitting does not give good prediction), but the lowest energy has already been found many times ($n\left( {\overline E } \right) > n_{{\mathrm{max}}}$, e.g., *n*_max_ = 1000). This happens for very easy problems. Or, if the fitting is consistently predicting another $E_{{\mathrm{min}}}^{{\mathrm{pred}}} \ne \overline E$, but $n\left( {\overline E } \right)$ is very large and ${\mathrm{\Gamma }} > {\mathrm{\Gamma }}^{{\mathrm{pred}}}\left( {\overline E - 1} \right)$, so according to the dynamics, it seems a lower energy should have been found already.

In such cases we exit the algorithm (Step 7) with the decision: $\overline E \ne E_{{\mathrm{min}}}^{{\mathrm{pred}}}$, $E_{{\mathrm{min}}}^{{\mathrm{dec}}} = \overline E$.

### Circulant matrices for the Ramsey number problem

Kalbfleisch^69^ argued that there should be coloring solutions of complete graphs for the Ramsey problem that can be described with a circulant form adjacency matrix (e.g., all red edges are 0-s, blue edges are 1-s in this matrix), or matrices that are close to such a circulant form. Although there is no formal proof of this statement, one expects this to be true also from the SAT formulation of the Ramsey problem. In the SAT formulation, the clauses have a very high degree of symmetry: all variables participate in the same way (4) in all the clauses, which run over all the possible *m*-cliques. This observation on symmetry can be exploited, allowing us to do part of the search in a much smaller space than the original space, where all the variables could in principle change independently from one another. More precisely, we first define a MaxSAT problem which has only *N* − 1 variables (instead of the full *N*(*N* − 1)/2 by choosing, e.g., those associated with the links of the first node: *x*_1_ = *a*_1,2_, *x*_2_ = *a*_1,3_, …, *x*_*N*−1_ = *a*_1,*N*_ as problem variables (here *a*_*i*,*j*_ denotes the adjacency matrix) and defining the variables of the links of the other nodes by the circular permutation of this vector ***x***, to obtain a circulant matrix (e.g., *a*_2,3_ = *a*_1,2_, *a*_*i*,*j*_ = *a*_*i*−1,*j*−1_). Taking the SAT form of the Ramsey problem we replace the variable of a link *a*_*i*,*j*_ with *x*_*j*−*i*_, thus reducing the number of independent variables from *N*(*N* − 1)/2 to *N* − 1. The number of clauses will also be reduced, because we can now eliminate the repeated ones. This way we obtain a much smaller MaxSAT problem, on which we apply our solver, and starting from random initial conditions we search for low-energy states, which are relatively easily found. We save the ***x*** vectors (the Boolean values) corresponding to such low-energy circulant matrix states. For *N* = 42 we have found circulant type matrices having only 6, 14, 20, 26, etc. monochromatic 5-cliques, indicating that they may already be close to a solution. After saving these circulant matrix states (with small number of monochromatic 5-cliques) we return to the original 10-SAT problem (with *N*(*N* − 1)/2 variables, without the symmetry constraint), and start a new trajectory from the corner of the hypercube corresponding to the saved matrix state, but now without symmetry restriction.

## Electronic supplementary material

Supplementary Information

Description of Additional Supplementary Files

Supplementary Data 1

Supplementary Data 2

Supplementary Data 3

Supplementary Data 4

## Data Availability

Datasets generated and/or analysed during the current study are available from the corresponding author on reasonable request. The source code for the analog MaxSAT solver can be accessed at https://github.com/molnarb14/analog-maxSAT-solver.
