# Supplementary material for: A continuous-time MaxSAT solver with high analog performance
Source: Nat Commun. 2018 Nov 19;9:4864. doi: 10.1038/s41467-018-07327-2 (PMC6242876; doi:10.1038/s41467-018-07327-2)
Supplement: Supplementary file 1 — Supplementary Information [file 41467_2018_7327_MOESM1_ESM.pdf]

Supplementary Information

A continuous-time Max-SAT solver with high analog performance

Molnár *et al.*

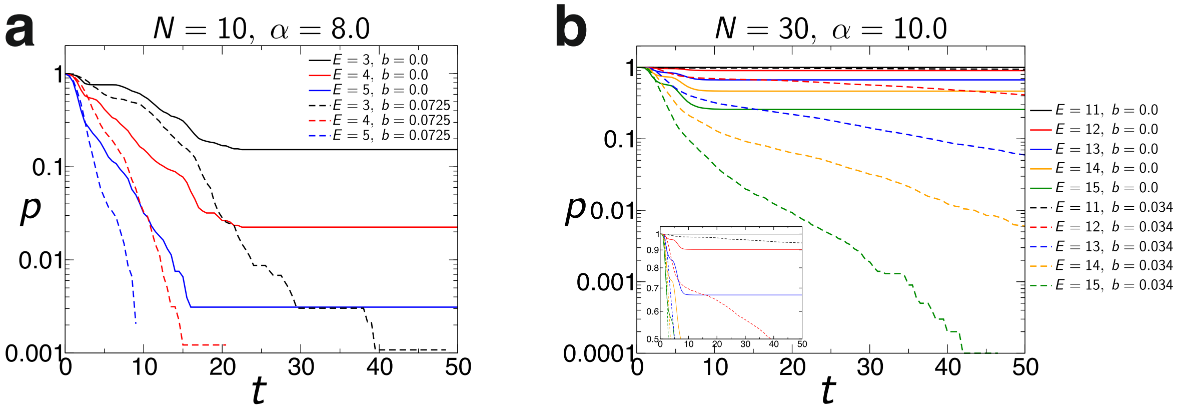

Supplementary Figure 1: **The** $p(E,t)$ **distribution of transient times compared between the SAT-solver algorithm presented in [1] and the new max-SAT solver dynamics.** $p(E,t)$ is the probability that up to time t a trajectory has not yet visited an orthant with energy smaller than E. As shown for two max-SAT instances **(a)** $N = 10$, $\alpha = 8.0$, $b = 0.0725$, $E_{\text{min}} = 3$. **(b)** $N = 30$, $\alpha = 10.0$, $b = 0.034$, $E_{\text{min}} = 11$, when $b = 0$ (the original analog SAT solver dynamics) the exponential decay of the distribution stops at relatively small t indicating that many trajectories have been trapped at $\boldsymbol{s} = 0$. When $b > 0$ the distribution is much steeper, and the trapping phenomenon does not occur.

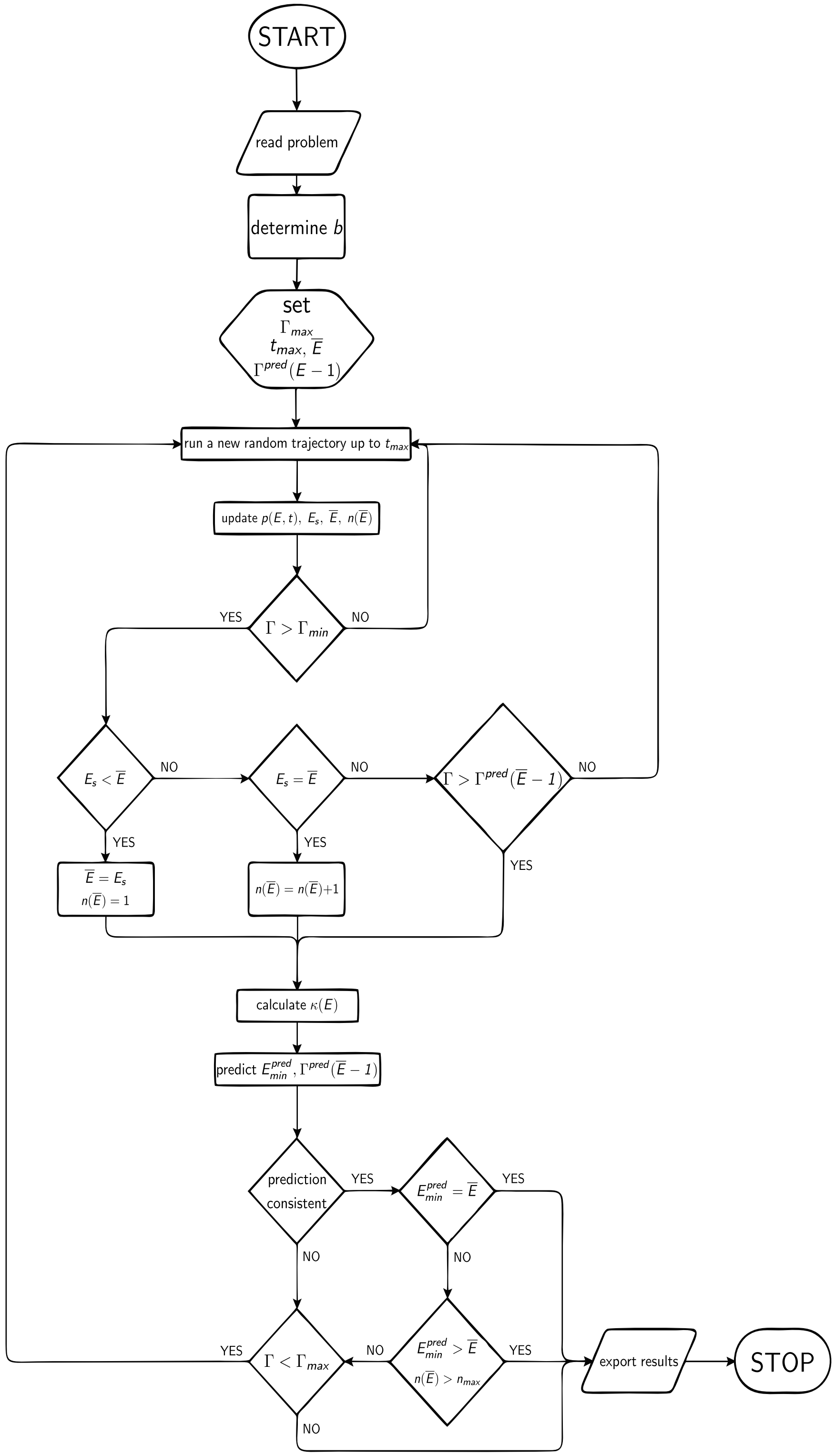

Supplementary Figure 2: **Flowchart of the dynamics described in detail in the Methods section**.

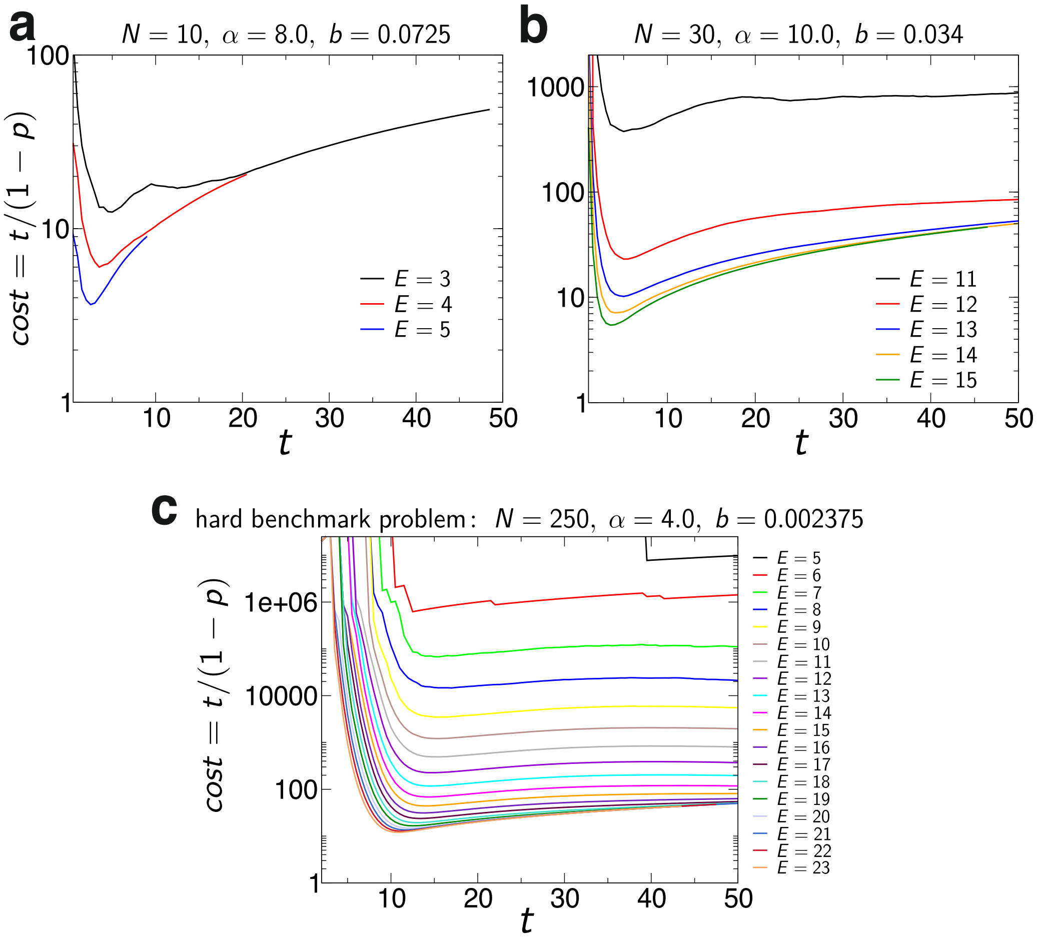

Supplementary Figure 3: **The cost of finding an energy level as function of time**. If we run Γ trajectories, each one up to time t, the total cost is proportional with Γt The number of times an energy level was found is equal to $(1 - p(E,t))\Gamma$, so we estimate the cost of finding a state with energy E as $t/(1 - p(E,t))$. We plot this cost function for different energy levels for three different MaxSAT instances. **(a)** $N = 10$, $\alpha = 8.0$, $b = 0.0725$, $E_{\min} = 3$. **(b)** $N = 30$, $\alpha = 10.0$, $b = 0.034$, $E_{\min} = 11$. **(c)** The hard benchmark problem presented in detail in Figs. 2, 4 of the main text with $N = 250$, $\alpha = 4.0$, $b = 0.002375$, $E_{\min} = 5$. The curves show a minimum at a relatively low t, indicating that it is more efficient to run many short trajectories, than running a few for longer times. In case of random SAT instances with $N \leq 100$ we usually choose $t_{\max} = 25$ For larger instances such as the benchmark problem we used $t_{\max} = 50$ in our Max-CTDS algorithm.

a

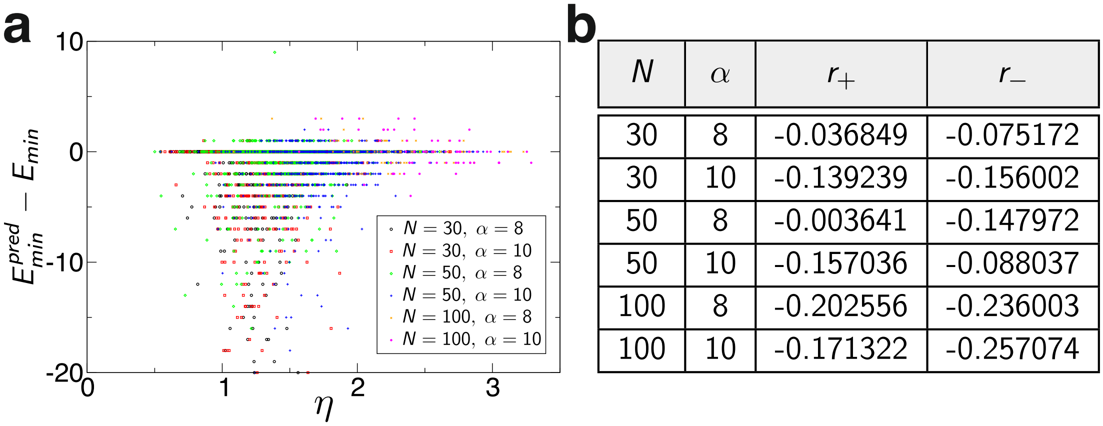

b

N	α	r_+	r_-
30	8	-0.036849	-0.075172
30	10	-0.139239	-0.156002
50	8	-0.003641	-0.147972
50	10	-0.157036	-0.088037
100	8	-0.202556	-0.236003
100	10	-0.171322	-0.257074

Supplementary Figure 4: **Correlation between the error of prediction and hardness of random 3-MaxSAT instances**. **(a)** The difference between the predicted and real global minimum value as function of the hardness measure $\eta = -\log\kappa/\log N$, which is applicable for individual instances. We use different symbols and colors for instances with different N and α (see legend). Large errors occur mainly in easy problems, at small η values. **(b)** This is also shown by the negative values of the Pearson correlation coefficients obtained between the absolute value of errors and η, calculated separately for positive (r_+) and negative errors (r_-).

a

Γ	$\overline{E}$	$n(\overline{E})$	E_0	E_{min}^{pred}	$\Gamma^{pred}(\overline{E}-1)$
**151**	**3**	**2**	**-0.5**	**0**	**730**
213	3	3	-1.8	0	586
**237**	**2**	**1**	**-3.3**	**0**	**1199**
...	...	...	...	...	...
9734	2	10	0.5	1	71428
9964	2	11	0.5	1	49582
**12129**	**2**	**12**	**0.4**	**1**	**54673**
33975	2	30	0.5	1	98940
...	...	...	...	...	...
40004	2	40	0.6	1	141636
...	...	...	...	...	...
56138	2	49	0.6	1	162964
**56333**	**2**	**50**	**0.6**	**1**	**162717**
56375	2	51	0.6	1	162384
...	...	...	...	...	...
292829	2	269	0.9	1	1995725
**294125**	**2**	**270**	**0.9**	**1**	**2000000**
294454	2	271	0.9	1	2000000
...	...	...	...	...	...
**1038118**	**2**	**1000**	**0.9**	**1**	

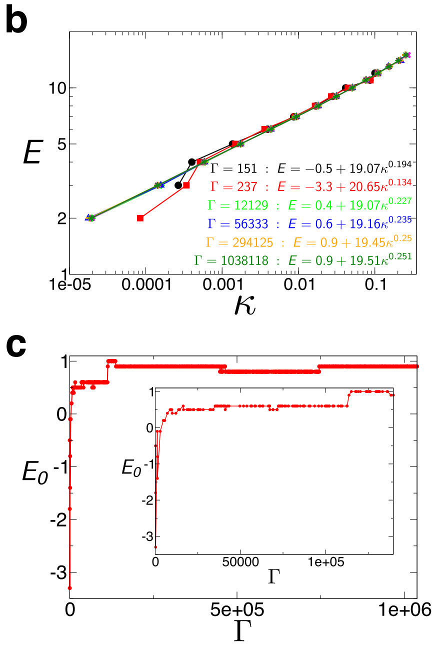

Supplementary Figure 5: **Performance of our algorithm on the 4-SAT problem HG-4SAT-V150-C1350-24.** The instance can be downloaded from [2]. Similarly, to Fig. 4 of the main text in (a) we show the values of the relevant measures at each fitting performed. The fitting for each colored line is shown in **(b)**. **(c)** The parameter E_0 that provides the final prediction of the global optimum is shown as function of Γ, the number of trajectories ran. In this case, our prediction slightly underestimates the minimum, predicting 1. However, after the energy state 2 is found 1000 times we stop the search.

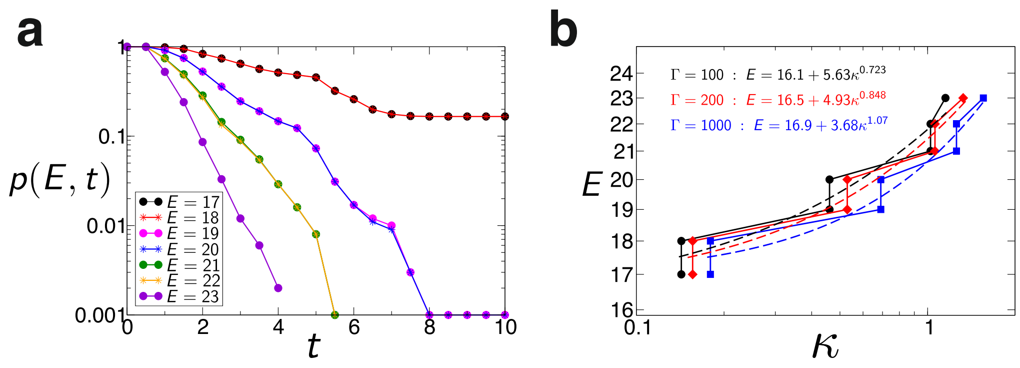

Supplementary Figure 6: **Predicting the global optimum in a spin-glass hard benchmark problem**. $k = 2$, N $= 27$, $M = 162$, $E_{\min} = 17$, $b = 0.042438$ (the problem instance t3pm3-5555.spn.cnf can be downloaded from [2]). Note, Max-2-SAT is also NP-hard, unlike 2-SAT, which is in P. **(a)** The $p(E,t)$distributions after running $\Gamma = 1000$ trajectories. Because of the special structure of the spin-glass problem pairs of consecutive energy levels show the same distributions (a single spin-flip can change the energy only in units of 2). **(b)** The energy E as function of the estimated $\kappa(E)$ values after running $\Gamma = 100, 200, 1000$ trajectories (see legend). The fitted curves are shown with dashed lines. $E_0 \in [16, 17)$ indicating a correct prediction already after $\Gamma = 100$ trajectories. This is an easy problem for our algorithm and $E_{\min} = 17$ is found 79 times by the first $\Gamma = 100$ trajectories.

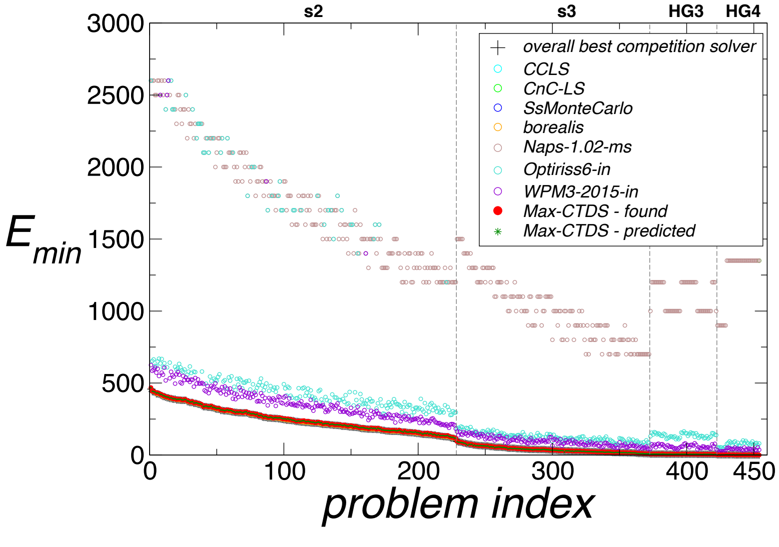

Supplementary Figure 7. **Algorithm performance on competition MaxSAT problems**. Using the 454 random benchmark problems from the 2016 SAT competition [2], we compared the minimum energy found by different incomplete solvers (legend) and the minimum found/predicted by our Max-CTDS. The black + symbols represent the overall best results by all the competition solvers. There are 4 categories of problems, separated by vertical dashed lines: s2 (Abrame-Habet) are Max 2-SAT with $N \in [100, 200]$ and $M \in [1200, 2600]$; s3 (Abrame-Habet) are Max 3-SAT with $N \in [70, 110]$ and $M \in [700, 1500]$; HG3 (high-girth) are Max 3-SAT problems with $N \in [250, 300]$ and $M \in [1000, 1200]$; HG4 are Max 4-SAT problems with $N \in [100, 150]$ and $M \in [900, 1350]$.

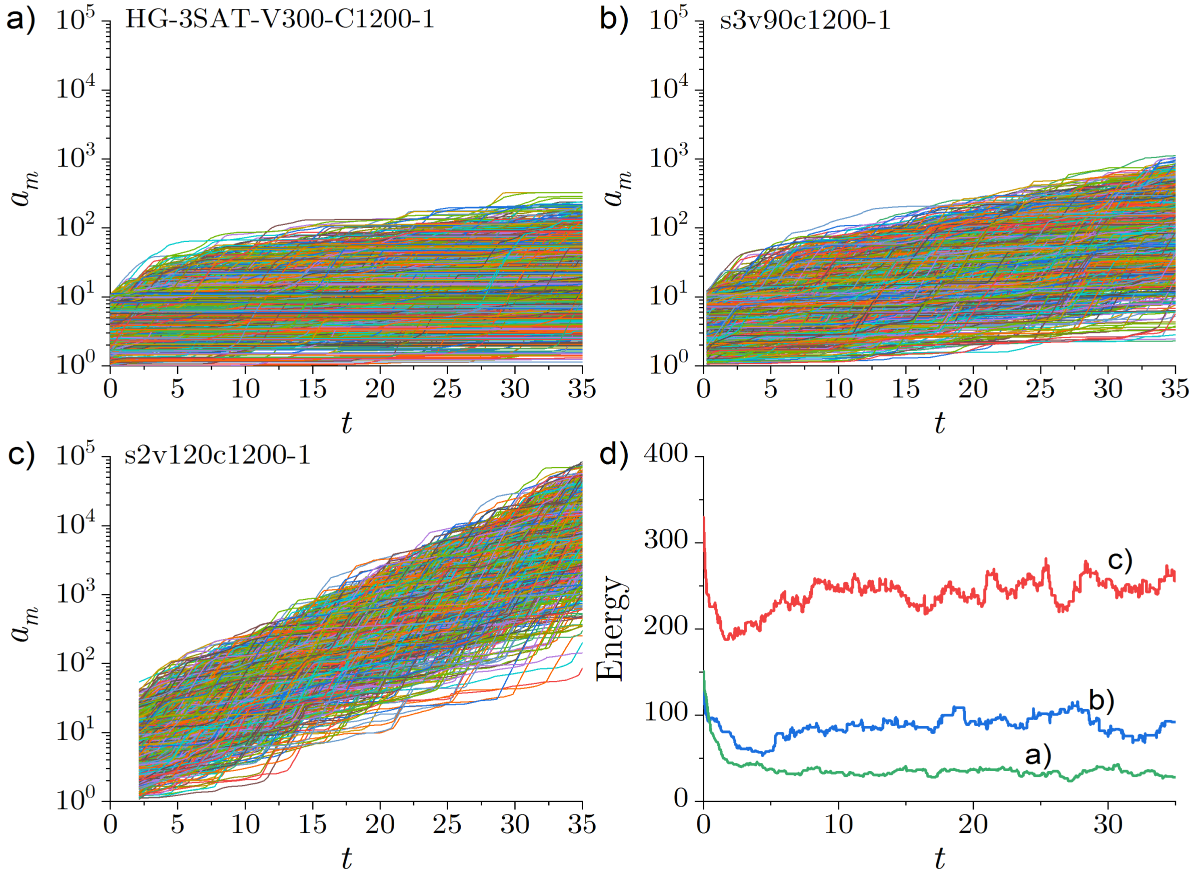

Supplementary Figure 8. **Stiffness of ODEs.** Three competition problems with the same $M = 1200$ total number of clauses but with different minimum energies: a) of 6, b) of 47 and c) of 161. d) Evolution of the energy for the three problems during the search dynamics.

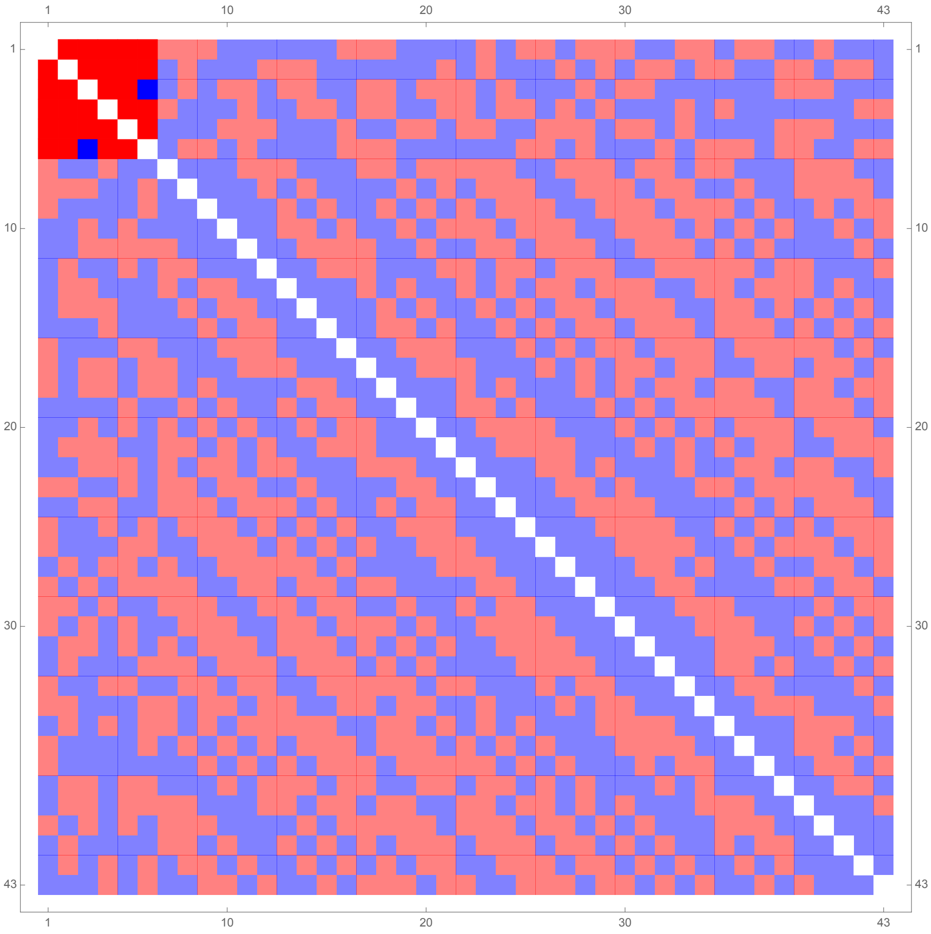

Supplementary Figure 9. **Reordered adjacency matrix for $N = 43$.** We reordered the nodes of the matrix from Fig. 7d such that the 6 nodes that included the two 5-cliques can be seen in the left corner.

Supplementary datasets

Supplementary Data 1. **The 3-SAT problem shown in Figure 1 of the main text.** We have $N = 10$ variables and $M = 80$ clauses (constraints) in conjunctive normal form. Each clause is shown in parenthesis as the series of 3 variables or their negation (indicated by negative sign). For example, the first clause indicates the constraint: $(x_5 \text{ OR } (\text{NOT } x_8) \text{ OR } x_9)$. Also provided as SupplementaryData1.cnf.

(5,-8,9)(-1,-3,-7)(9,4,-8)(-1,-9,4)(7,2,3)(9,5,4)(8,9,-3)(10,-5,9)(9,7,8)(3,1,6) (7,10,3)
(-5,10,-3)(-7,6,4)(-8,1,-10)(-1,-2,3)(-9,-2,-3)(5,7,8)(-5,-3,4)(9,-2,1)(-3,-1,-7)(10,5,4)
(-7,-10,-4)(-9,-10,3)(2,-1,10)(-5,-10,-7)(-9,6,8)(-9,-4,-8)(-5,-3,-8)(-9,3,-7)(-6,2,5)
(-2,1,-8)(1,6,9)(5,-9,2)(10,-1,7)(5,-1,-3)(6,-7,2)(8,-5,7)(-8,-7,-3)(4,-7,3)(4,-9,2)(1,6,-7)
(-9,-2,5)(10,-4,-5)(4,-2,-9)(-7,2,1)(4,2,-8)(-2,-10,-5)(6,-3,7)(-1,-3,7)(-1,6,4)(-9,-4,3)
(-4,10,-5)(9,6,-2)(-8,-2,5)(2,-1,3)(-6,-4,10)(7,-5,2)(7,3,-5)(-7,9,-6)(-4,6,2)(-6,9,-5)
(-10,-1,2)(5,-8,-7)(8,7,-2)(-8,-2,1)(6,1,-8)(8,5,-2)(-8,3,6)(10,2,-3)(9,-7,2)(-6,10,2)
(1,-3,4)(6,2,-8)(9,2,10)(2,5,-1)(-1,8,4)(-3,1,-4)(-10,9,-7)(-4,-5,-9)(-6,-7,10)

Supplementary Data 2. **The optimal solution found for the HG-3SAT-V250-C1000-1.cnf MaxSAT competition problem:** $E_{\min} = 5$. The sign of the variables indicate whether their value is True (positive sign) or False (negative sign). Also provided as SupplementaryData2.txt.

-1 2 3 4 5 -6 7 8 -9 10 11 12 13 14 -15 -16 -17 18 -19 -20 -21 -22 -23 24 25 26 27 28
29 30 -31 32 33 -34 -35 -36 -37 38 39 -40 -41 42 -43 -44 -45 -46 -47 48 49 50 -51 -52
53 54 55 -56 57 58 59 60 61 -62 -63 64 65 -66 -67 -68 -69 -70 71 -72 -73 -74 -75 76
77 -78 79 -80 81 -82 -83 -84 85 86 87 -88 -89 90 -91 -92 -93 94 95 96 97 -98 99 -100
-101 -102 103 -104 -105 -106 -107 -108 -109 -110 -111 -112 -113 -114 -115 116 117
-118 -119 120 -121 -122 123 124 -125 126 -127 -128 -129 -130 131 132 133 -134
-135 -136 137 -138 -139 140 -141 142 143 -144 -145 -146 147 148 149 -150 -151
-152 153 -154 -155 156 157 158 -159 160 161 -162 163 164 165 -166 -167 -168 169
-170 -171 -172 -173 174 -175 176 177 178 -179 180 181 182 183 184 -185 186 -187
188 -189 -190 -191 -192 193 -194 -195 196 197 -198 -199 200 -201 -202 203 204 205
-206 -207 208 209 210 211 212 -213 -214 215 216 -217 -218 219 -220 221 -222 223
-224 225 -226 -227 -228 -229 -230 231 -232 233 -234 235 236 -237 -238 239 -240
-241 -242 243 -244 245 -246 247 -248 -249 250

Supplementary Data 3. **The matrix shown in Fig. 7b of the main text that gives a complete Ramsey coloring of a graph with 42 nodes.** There are no monochromatic 5-cliques. ($E_{\min} = 0$) Also provided as SupplementaryData3.txt.

0 0 1 1 1 0 0 0 0 0 0 0 1 1 1 0 1 1 0 1 1 0 1 1 1 0 1 1 0 1 1 1 0 0 0 0 0 0 0 1 1 0
0 0 0 1 1 1 0 0 0 0 0 0 0 1 1 1 0 1 1 0 1 1 1 1 1 1 0 1 1 0 1 1 1 0 0 0 0 0 0 0 1 1
1 0 0 0 1 1 1 0 0 0 0 0 0 0 1 1 1 0 1 1 0 1 1 1 0 1 1 0 1 1 0 1 1 1 0 0 0 0 0 0 0 1
1 1 0 0 0 1 1 1 0 0 0 0 0 0 0 1 1 1 0 1 1 0 1 1 0 0 1 1 0 1 1 0 1 1 1 0 0 0 1 0 0 0
1 1 1 0 0 0 1 1 1 0 0 0 0 0 0 0 1 1 1 0 1 1 0 1 1 0 0 1 1 0 1 1 0 1 1 1 0 0 0 0 0 0
0 1 1 1 0 0 0 1 1 1 0 0 0 0 0 0 0 1 1 1 0 1 1 0 1 1 1 1 1 1 0 1 1 0 1 1 1 0 0 0 0 0
0 0 1 1 1 0 0 0 1 1 1 0 0 0 0 0 0 0 1 1 1 0 1 1 0 1 1 1 1 1 1 0 1 1 0 1 1 1 0 0 0 0
0 0 0 1 1 1 0 0 0 1 1 1 0 0 0 0 0 0 0 1 1 1 0 1 1 0 1 1 0 0 1 1 0 1 1 0 1 1 1 0 0 0
0 0 0 0 1 1 1 0 0 0 1 1 1 0 0 0 0 0 0 0 1 1 1 0 1 1 0 1 1 0 0 1 1 0 1 1 0 1 1 1 0 0
0 0 0 0 0 1 1 1 0 0 0 1 1 1 0 0 0 0 0 0 0 1 1 1 0 1 1 0 1 1 0 1 0 1 0 1 1 0 1 1 1 0
0 0 0 0 0 0 1 1 1 0 0 0 1 1 1 0 0 0 0 0 0 0 1 1 1 0 1 1 0 1 1 0 1 1 1 0 1 1 0 1 1 1
0 0 0 0 0 0 0 1 1 1 0 0 0 1 1 1 0 0 0 0 0 0 0 1 1 1 0 1 1 0 1 1 0 0 1 1 0 1 1 0 1 1
1 0 0 0 0 0 0 0 1 1 1 0 0 0 1 1 1 0 0 0 0 0 0 0 1 1 1 0 1 1 0 1 1 0 0 1 1 0 1 1 0 1
1 1 0 0 0 0 0 0 0 1 1 1 0 0 0 1 1 1 0 0 0 0 0 0 0 1 1 1 0 1 1 0 1 1 0 1 1 1 0 1 1 0
1 1 1 0 0 0 0 0 0 0 1 1 1 0 0 0 1 1 1 0 0 0 0 0 0 0 1 1 1 0 1 1 0 1 1 1 1 1 1 0 1 1
0 1 1 1 0 0 0 0 0 0 0 1 1 1 0 0 0 1 1 1 0 0 0 0 0 0 1 1 1 1 0 1 1 0 1 1 1 0 1 1 0 1
1 0 1 1 1 0 0 0 0 0 0 0 1 1 1 0 0 0 1 1 1 0 0 0 0 0 0 0 1 1 1 0 1 1 0 1 1 0 0 1 1 0
1 1 0 1 1 1 0 0 0 0 0 0 0 1 1 1 0 0 0 1 1 1 0 0 0 0 0 0 0 1 1 1 0 1 1 0 1 1 0 0 1 1
0 1 1 0 1 1 1 0 0 0 0 0 0 0 1 1 1 0 0 0 1 1 1 0 0 0 0 0 0 0 1 1 1 0 1 1 0 1 1 1 1 1
1 0 1 1 0 1 1 1 0 0 0 0 0 0 0 1 1 1 0 0 0 1 1 1 0 0 0 0 0 0 0 1 1 1 0 1 1 0 1 1 1 1
1 1 0 1 1 0 1 1 1 0 0 0 0 0 0 0 1 1 1 0 0 0 1 1 1 0 0 0 0 0 0 0 1 1 1 0 1 1 0 1 1 0
0 1 1 0 1 1 0 1 1 1 0 0 0 0 0 0 0 1 1 1 0 0 0 1 1 1 0 0 0 0 0 0 0 1 1 1 0 1 1 0 1 1

1 1 1 1 0 1 1 0 1 1 1 0 0 0 0 0 0 0 1 1 1 0 0 0 1 1 1 0 0 0 0 0 0 0 1 1 1 0 1 1 0 1
1 1 1 1 1 0 1 1 0 1 1 1 0 0 0 0 0 0 0 1 1 1 0 0 0 1 1 1 0 0 0 0 0 0 0 1 1 1 0 1 1 0
1 1 0 0 1 1 0 1 1 0 1 1 1 0 0 0 0 0 0 0 1 1 1 0 0 0 1 1 1 0 0 0 0 0 0 0 1 1 1 0 1 1
0 1 1 0 0 1 1 0 1 1 0 1 1 1 0 0 0 0 0 0 0 1 1 1 0 0 0 1 1 1 0 0 0 0 0 0 0 1 1 1 0 1
1 0 1 1 0 1 1 1 0 1 1 0 1 1 1 0 0 0 0 0 0 0 1 1 1 0 0 0 1 1 1 0 0 0 0 0 0 0 1 1 1 0
1 1 0 1 1 1 1 1 1 0 1 1 0 1 1 1 0 0 0 0 0 0 0 1 1 1 0 0 0 1 1 1 0 0 0 0 0 0 0 1 1 1
0 1 1 0 1 1 1 0 1 1 0 1 1 0 1 1 1 0 0 0 0 0 0 0 1 1 1 0 0 0 1 1 1 0 0 0 0 0 0 0 1 1
1 0 1 1 0 1 1 0 0 1 1 0 1 1 0 1 1 1 0 0 0 0 0 0 0 1 1 1 0 0 0 1 1 1 0 0 0 0 0 0 0 1
1 1 0 1 1 0 1 1 0 0 1 1 0 1 1 0 1 1 1 0 0 0 0 0 0 0 1 1 1 0 0 0 1 1 1 0 0 0 0 0 0 0
1 1 1 0 1 1 0 1 1 1 0 1 1 0 1 1 0 1 1 1 0 0 0 0 0 0 0 1 1 1 0 0 0 1 1 1 0 0 0 0 0 0
0 1 1 1 0 1 1 0 1 0 1 0 1 1 0 1 1 0 1 1 1 0 0 0 0 0 0 0 1 1 1 0 0 0 1 1 1 0 0 0 0 0
0 0 1 1 1 0 1 1 0 1 1 0 0 1 1 0 1 1 0 1 1 1 0 0 0 0 0 0 0 1 1 1 0 0 0 1 1 1 0 0 0 0
0 0 0 1 1 1 0 1 1 0 1 1 0 0 1 1 0 1 1 0 1 1 1 0 0 0 0 0 0 0 1 1 1 0 0 0 1 1 1 0 0 0
0 0 0 0 1 1 1 0 1 1 0 1 1 1 1 1 1 0 1 1 0 1 1 1 0 0 0 0 0 0 0 1 1 1 0 0 0 1 1 1 0 0
0 0 0 0 0 1 1 1 0 1 1 0 1 1 1 1 1 1 0 1 1 0 1 1 1 0 0 0 0 0 0 0 1 1 1 0 0 0 1 1 1 0
0 0 0 0 0 0 1 1 1 0 1 1 0 1 1 0 0 1 1 0 1 1 0 1 1 1 0 0 0 0 0 0 0 1 1 1 0 0 0 1 1 1
0 0 0 1 0 0 0 1 1 1 0 1 1 0 1 1 0 0 1 1 0 1 1 0 1 1 1 0 0 0 0 0 0 0 1 1 1 0 0 0 1 1
1 0 0 0 0 0 0 0 1 1 1 0 1 1 0 1 1 0 1 1 1 0 1 1 0 1 1 1 0 0 0 0 0 0 0 1 1 1 0 0 0 1
1 1 0 0 0 0 0 0 0 1 1 1 0 1 1 0 1 1 1 1 1 1 0 1 1 0 1 1 1 0 0 0 0 0 0 0 1 1 1 0 0 0
0 1 1 0 0 0 0 0 0 0 1 1 1 0 1 1 0 1 1 1 0 1 1 0 1 1 0 1 1 1 0 0 0 0 0 0 0 1 1 1 0 0

Supplementary Data 4. **The coloring matrix shown in Fig. 7d with only 2 monochromatic 5-cliques sitting on 6 vertices of a complete graph with 43 nodes ($E_{\min} = 2$).** Also provided as SupplementaryData4.txt.

0 1 1 1 0 0 0 0 1 1 1 0 1 0 1 0 0 1 0 0 0 1 1 0 0 0 1 0 0 1 0 1 0 1 1 1 0 0 0 0 1 1 1
1 0 1 1 1 0 0 1 0 1 1 1 0 1 0 1 0 0 1 0 0 0 1 1 0 0 0 1 0 0 1 0 1 0 1 1 1 0 0 0 0 1 1
1 1 0 1 1 1 0 0 1 0 1 1 1 0 1 0 1 0 0 1 0 0 0 1 1 0 0 0 1 0 0 1 0 1 0 1 1 1 0 1 0 0 1
1 1 1 0 1 1 1 0 0 1 0 1 1 1 0 1 0 1 0 0 1 0 0 0 1 1 0 0 0 1 0 0 1 0 1 0 1 1 1 0 1 0 0
0 1 1 1 0 1 1 1 0 0 0 0 1 1 1 0 1 0 1 0 0 1 0 0 0 1 1 0 0 0 1 0 0 1 0 1 0 1 1 1 0 1 0
0 0 1 1 1 0 1 1 1 0 0 0 0 1 1 1 0 1 0 1 0 0 1 0 0 0 1 1 0 0 0 1 0 0 1 0 1 0 1 1 1 0 0
0 0 0 1 1 1 0 1 1 1 0 0 0 0 1 1 1 0 1 0 1 0 0 1 0 0 0 1 1 0 0 0 1 0 0 1 0 1 0 1 1 1 0
0 1 0 0 1 1 1 0 1 1 1 0 0 1 0 1 1 1 0 1 0 1 0 0 1 0 0 0 1 1 0 0 0 1 0 0 1 0 1 0 1 1 1
1 0 1 0 0 1 1 1 0 1 1 1 0 0 1 0 1 1 1 0 1 0 1 0 0 1 0 0 0 1 1 0 0 0 1 0 0 1 0 1 0 1 1
1 1 0 1 0 0 1 1 1 0 1 1 1 0 0 1 0 1 1 1 0 1 0 1 0 0 1 0 0 0 1 1 0 0 0 1 0 0 1 0 1 0 1
1 1 1 0 0 0 0 1 1 1 0 1 1 1 0 0 0 0 1 1 1 0 1 0 1 0 0 1 0 0 0 1 1 0 0 0 1 0 0 1 0 1 0
0 1 1 1 0 0 0 0 1 1 1 0 1 1 1 0 0 0 0 1 1 1 0 1 0 1 0 0 1 0 0 0 1 1 0 0 0 1 0 0 1 0 1
1 0 1 1 1 0 0 0 0 1 1 1 0 1 1 1 0 0 1 0 1 1 1 0 1 0 1 0 0 1 0 0 0 1 1 0 0 0 1 0 0 1 0
0 1 0 1 1 1 0 1 0 0 1 1 1 0 1 1 1 0 0 1 0 1 1 1 0 1 0 1 0 0 1 0 0 0 1 1 0 0 0 1 0 0 1
1 0 1 0 1 1 1 0 1 0 0 1 1 1 0 1 1 1 0 0 1 0 1 1 1 0 1 0 1 0 0 1 0 0 0 1 1 0 0 0 1 0 0
0 1 0 1 0 1 1 1 0 1 0 0 1 1 1 0 1 1 1 0 0 0 0 1 1 1 0 1 0 1 0 0 1 0 0 0 1 1 0 0 0 1 0
0 0 1 0 1 0 1 1 1 0 0 0 0 1 1 1 0 1 1 1 0 0 0 0 1 1 1 0 1 0 1 0 0 1 0 0 0 1 1 0 0 0 1
1 0 0 1 0 1 0 1 1 1 0 0 0 0 1 1 1 0 1 1 1 0 0 1 0 1 1 1 0 1 0 1 0 0 1 0 0 0 1 1 0 0 0
0 1 0 0 1 0 1 0 1 1 1 0 1 0 0 1 1 1 0 1 1 1 0 0 1 0 1 1 1 0 1 0 1 0 0 1 0 0 0 1 1 0 0

0 0 1 0 0 1 0 1 0 1 1 1 0 1 0 0 1 1 1 0 1 1 1 0 0 1 0 1 1 1 0 1 0 1 0 0 1 0 0 0 1 1 0
0 0 0 1 0 0 1 0 1 0 1 1 1 0 1 0 0 1 1 1 0 1 1 1 0 0 0 0 1 1 1 0 1 0 1 0 0 1 0 0 0 1 1
1 0 0 0 1 0 0 1 0 1 0 1 1 1 0 0 0 0 1 1 1 0 1 1 1 0 0 0 0 1 1 1 0 1 0 1 0 0 1 0 0 0 1
1 1 0 0 0 1 0 0 1 0 1 0 1 1 1 0 0 0 0 1 1 1 0 1 1 1 0 0 0 0 1 1 1 0 1 0 1 0 0 1 0 0 0
0 1 1 0 0 0 1 0 0 1 0 1 0 1 1 1 0 1 0 0 1 1 1 0 1 1 1 0 0 1 0 1 1 1 0 1 0 1 0 0 1 0 0
0 0 1 1 0 0 0 1 0 0 1 0 1 0 1 1 1 0 1 0 0 1 1 1 0 1 1 1 0 0 1 0 1 1 1 0 1 0 1 0 0 1 0
0 0 0 1 1 0 0 0 1 0 0 1 0 1 0 1 1 1 0 1 0 0 1 1 1 0 1 1 1 0 0 1 0 1 1 1 0 1 0 1 0 0 1
1 0 0 0 1 1 0 0 0 1 0 0 1 0 1 0 1 1 1 0 0 0 0 1 1 1 0 1 1 1 0 0 0 0 1 1 1 0 1 0 1 0 0
0 1 0 0 0 1 1 0 0 0 1 0 0 1 0 1 0 1 1 1 0 0 0 0 1 1 1 0 1 1 1 0 0 0 0 1 1 1 0 1 0 1 0
0 0 1 0 0 0 1 1 0 0 0 1 0 0 1 0 1 0 1 1 1 0 0 0 0 1 1 1 0 1 1 1 0 0 1 0 1 1 1 0 1 0 1
1 0 0 1 0 0 0 1 1 0 0 0 1 0 0 1 0 1 0 1 1 1 0 1 0 0 1 1 1 0 1 1 1 0 0 1 0 1 1 1 0 1 0
0 1 0 0 1 0 0 0 1 1 0 0 0 1 0 0 1 0 1 0 1 1 1 0 1 0 0 1 1 1 0 1 1 1 0 0 1 0 1 1 1 0 1
1 0 1 0 0 1 0 0 0 1 1 0 0 0 1 0 0 1 0 1 0 1 1 1 0 1 0 0 1 1 1 0 1 1 1 0 0 0 0 1 1 1 0
0 1 0 1 0 0 1 0 0 0 1 1 0 0 0 1 0 0 1 0 1 0 1 1 1 0 0 0 0 1 1 1 0 1 1 1 0 0 0 0 1 1 1
1 0 1 0 1 0 0 1 0 0 0 1 1 0 0 0 1 0 0 1 0 1 0 1 1 1 0 0 0 0 1 1 1 0 1 1 1 0 0 1 0 1 1
1 1 0 1 0 1 0 0 1 0 0 0 1 1 0 0 0 1 0 0 1 0 1 0 1 1 1 0 1 0 0 1 1 1 0 1 1 1 0 0 1 0 1
1 1 1 0 1 0 1 0 0 1 0 0 0 1 1 0 0 0 1 0 0 1 0 1 0 1 1 1 0 1 0 0 1 1 1 0 1 1 1 0 0 1 0
0 1 1 1 0 1 0 1 0 0 1 0 0 0 1 1 0 0 0 1 0 0 1 0 1 0 1 1 1 0 1 0 0 1 1 1 0 1 1 1 0 0 0
0 0 1 1 1 0 1 0 1 0 0 1 0 0 0 1 1 0 0 0 1 0 0 1 0 1 0 1 1 1 0 0 0 0 1 1 1 0 1 1 1 0 0
0 0 0 1 1 1 0 1 0 1 0 0 1 0 0 0 1 1 0 0 0 1 0 0 1 0 1 0 1 1 1 0 0 0 0 1 1 1 0 1 1 1 0
0 0 1 0 1 1 1 0 1 0 1 0 0 1 0 0 0 1 1 0 0 0 1 0 0 1 0 1 0 1 1 1 0 1 0 0 1 1 1 0 1 1 1
1 0 0 1 0 1 1 1 0 1 0 1 0 0 1 0 0 0 1 1 0 0 0 1 0 0 1 0 1 0 1 1 1 0 1 0 0 1 1 1 0 1 1
1 1 0 0 1 0 1 1 1 0 1 0 1 0 0 1 0 0 0 1 1 0 0 0 1 0 0 1 0 1 0 1 1 1 0 1 0 0 1 1 1 0 1
1 1 1 0 0 0 0 1 1 1 0 1 0 1 0 0 1 0 0 0 1 1 0 0 0 1 0 0 1 0 1 0 1 1 1 0 0 0 0 1 1 1 0

Supplementary Note

Supplementary Note 1: **Stiffness of ODEs.** The major issue with running ODEs such as our CTDS solver on digital machines is the appearance of stiffness (https://en.wikipedia.org/wiki/Stiff equation). This is the phenomenon when the discretization becomes numerically unstable and the integrator is forced to take smaller and smaller stepsize (in the time variable t, i.e., $t + \Delta t$ for the next step) in order to keep the errors of the calculation within prescribed bounds. Thus, the integration starts spending a large number of discrete steps, but the evolution of the continuous analog time t and thus also that of the trajectory, start to slow down rapidly, and essentially the "integration freezes out." Mathematically, a system is stiff if it contains bounded variables (in our case the $s_i \in [-1,1]$) and very (exponentially) large rates of change for those variables (in our case ds_i/dt). Stiffness can be handled to some extent using implicit integration methods instead of explicit ones as they have a larger domain of integration stability than explicit methods, however, they will also freeze out, eventually. In our case the integration becomes increasingly stiff over time, due to the exponentially increasing separation of the values of the auxiliary variables (which drive the large values for the velocities ds_i/dt). The rate at which stiffness appears depends on the nature of the MaxSAT problem. For problems for which the optimum lowest number of unsat clauses (minimum energy value) is fairly large, i.e, $O(M)$ (where $M$is the total number of clauses),

stiffness will appear faster than for problems with a low minimum energy value, i.e., of $O(1)$. In particular, the Max 2-SAT problems s2 in the 2016 Max-SAT competition almost all have a large value for the minimum (optimum) energy value, compared to the total number of clauses, and thus the solver experiences faster separation of the auxiliary variables than for the other problems as shown in Supplemenary Figure 8. Because of this, the performance is slightly behind those of the competition solvers for these s2 types of problems. Supplementary Figure 8 presents three MaxSAT 2016 competition problems, all with $M = 1200$ clauses: a) a HG Max 3-SAT problem with lowest energy of 6, b) an s3 Max 3-SAT problem with minimum energy of 47 and c) an **s2** Max 2-SAT problem with a minimum energy of 161, all on the same scales. One can see that for the **s2** problem the auxiliary variables grow much faster than for the other, slowing the search dynamics due to stiffness. Supplementary Figure 8d) shows the evolution of the energy values as function of time during the dynamics for the three problems, illustrating that in the case of the s2 problem there are many more clauses in violation than for the other two, generating the faster increase of the auxiliary variables, shown in c). The fact that our CTDS solver still finds very close solutions even for these problems it shows that it has a "smart" search dynamics. However, we would like to emphasize, that once implemented in hardware, the current flow or voltage behavior of the physical circuit directly correspond to the equations of the solver and these numerical integration issues will not occur.

Supplementary References

[1] Ercsey-Ravasz, M. & Toroczkai, Z. Optimization hardness as transient chaos in an analog approach to constraint satisfaction. *Nature Physics* **7**, 966-970 (2011)

[2] Benchmarks. Eleventh Evaluation of Max-SAT Solvers. http://www.maxsat.udl.cat/16/benchmarks/index.html (2016).
