# Supplementary material for: A continuous-time MaxSAT solver with high analog performance
Source: Nat Commun. 2018 Nov 19;9:4864. doi: 10.1038/s41467-018-07327-2 (PMC6242876; doi:10.1038/s41467-018-07327-2)
Supplement: Supplementary file 2 — Description of Additional Supplementary Files [file 41467_2018_7327_MOESM2_ESM.pdf]

Description of additional supplementary files

File name: Supplementary Data 1
Description: The 3-SAT problem shown in Figure 1 of the main text. We have $N = 10$ variables and $M = 80$ clauses (constraints) in conjunctive normal form. Each clause is shown on a separate line as the series of 3 variables or their negation (indicated by negative sign). For example, the first clause indicates the constraint: $(x_5 \text{ OR } (\text{NOT } x_8) \text{ OR } x_9)$.

File name: Supplementary Data 2
Description: The optimal solution found for the HG-3SAT-V250-C1000-1.cnf MaxSAT competition problem: $E_{\text{min}} = 5$. The sign of the variables indicate whether their value is True (positive sign) or False (negative sign).

File name: Supplementary Data 3
Description: The matrix shown in Fig. 7b of the main text that gives a complete Ramsey coloring of a graph with 42 nodes. There are no monochromatic 5-cliques. ($E_{\text{min}} = 0$)

File name: Supplementary Data 4
Description: The coloring matrix shown in Fig. 7d with only 2 monochromatic 5-cliques sitting on 6 vertices of a complete graph with 43 nodes ($E_{\text{min}} = 2$).
